# Deep-Sea Sponges and Corals off the Western Coast of Florida—Intracellular Mechanisms of Action of Bioactive Compounds and Technological Advances Supporting the Drug Discovery Pipeline

**DOI:** 10.3390/md21120615

**Published:** 2023-11-28

**Authors:** Mina Iskandar, Kira M. Ruiz-Houston, Steven D. Bracco, Sami R. Sharkasi, Cecilia L. Calabi Villarroel, Meghna N. Desai, Alexandra G. Gerges, Natalia A. Ortiz Lopez, Miguel Xiao Barbero, Amelia A. German, Vinoothna S. Moluguri, Selina M. Walker, Juliana Silva Higashi, Justin M. Palma, Daena Z. Medina, Miit Patel, Prachi Patel, Michaela Valentin, Angelica C. Diaz, Jonathan P. Karthaka, Atzin D. Santiago, Riley B. Skiles, Luis A. Romero Umana, Maxwell D. Ungrey, Anya Wojtkowiak, Domenica V. Howard, Remy Nurge, Katharine G. Woods, Meera Nanjundan

**Affiliations:** Department of Molecular Biosciences, University of South Florida, 4202 East Fowler Avenue, ISA2015, Tampa, FL 33620, USA; minaiskandar@usf.edu (M.I.); kiraruizhouston@usf.edu (K.M.R.-H.); stevenbracco@usf.edu (S.D.B.); samisharkasi@usf.edu (S.R.S.); ceciliac@usf.edu (C.L.C.V.); meghnadesai1@usf.edu (M.N.D.); alexandragaylegerges@usf.edu (A.G.G.); nataliaortizlopez@usf.edu (N.A.O.L.); miguelxiaobarbero@usf.edu (M.X.B.); aagerman@usf.edu (A.A.G.); vinoothna@usf.edu (V.S.M.); selina5@usf.edu (S.M.W.); julianasilva@usf.edu (J.S.H.); justinmichaelpalma@usf.edu (J.M.P.); dmedina4@usf.edu (D.Z.M.); miitpatel@usf.edu (M.P.); patel24@usf.edu (P.P.); michaelav408@usf.edu (M.V.); adiaz3@usf.edu (A.C.D.); jonathan83@usf.edu (J.P.K.); atzindtzoara@usf.edu (A.D.S.); rbskiles@usf.edu (R.B.S.); laromero@usf.edu (L.A.R.U.); maxwellungrey@usf.edu (M.D.U.); wojtkowiak@usf.edu (A.W.); dvhoward@usf.edu (D.V.H.); knurge@usf.edu (R.N.); katharinew@usf.edu (K.G.W.)

**Keywords:** marine sponges, marine corals, deep sea, western coast of Florida, NOAA, cytoskeleton dynamics, protein trafficking, protein signaling, drug discovery, aquaculture, in vitro culturing

## Abstract

The majority of natural products utilized to treat a diverse array of human conditions and diseases are derived from terrestrial sources. In recent years, marine ecosystems have proven to be a valuable resource of diverse natural products that are generated to defend and support their growth. Such marine sources offer a large opportunity for the identification of novel compounds that may guide the future development of new drugs and therapies. Using the National Oceanic and Atmospheric Administration (NOAA) portal, we explore deep-sea coral and sponge species inhabiting a segment of the U.S. Exclusive Economic Zone, specifically off the western coast of Florida. This area spans ~100,000 km^2^, containing coral and sponge species at sea depths up to 3000 m. Utilizing PubMed, we uncovered current knowledge on and gaps across a subset of these sessile organisms with regards to their natural products and mechanisms of altering cytoskeleton, protein trafficking, and signaling pathways. Since the exploitation of such marine organisms could disrupt the marine ecosystem leading to supply issues that would limit the quantities of bioactive compounds, we surveyed methods and technological advances that are necessary for sustaining the drug discovery pipeline including in vitro aquaculture systems and preserving our natural ecological community in the future. Collectively, our efforts establish the foundation for supporting future research on the identification of marine-based natural products and their mechanism of action to develop novel drugs and therapies for improving treatment regimens of human conditions and diseases.

## 1. Introduction

### 1.1. Marine Corals and Sponges, the Supply Issue, and Marine Ecosystem Preservation

Although marine organisms occupy a large area of the Earth’s surface, only 5% have been investigated with respect to their naturally produced bioactive metabolites [1]. Thus, there remains a large gap for identifying many more novel agents with therapeutic potential. According to a 2022 study, the bioactivities of secondary metabolites from corals include anti-tumor, anti-fungal, and anti-viral activities, amongst others [1]. Likewise, the bioactivities from marine sponges are also well established [2].

For clinical applications, it becomes necessary to scale up the production of these marine metabolites for generating a continuous and adequate quantity of the final product. It is estimated that 50 mg of a bioactive compound is required for the “hit-to-lead” process with another 50 mg needed for assessing the pharmacokinetics of ADMET (adsorption, distribution, metabolism, excretion, and toxicity) together with in vivo pre-clinical testing, which itself may require ~2000 mg [3]. Unfortunately, such clinical testing requirements for kilogram-scale quantities [1] are difficult to meet due to the “supply problem” [4]. This term refers to the low abundance of native metabolites per gram of marine tissue, together with the immense destruction to the natural population to acquire sufficient quantities if obtained from natural resources [4].

Along with the events surrounding global climate change, including pollution, overfishing, and destructive environmental conditions (e.g., coral bleaching—the disruption of the symbiotic interaction between corals and their symbiont dinoflagellates [5]), the protection of these marine organisms within their native environment has become increasingly important [6]. Such environmental factors are known to alter the quality and quantity of metabolites due to diminishing marine invertebrate survivability, leading to insufficient biomass [7]. Due to the fact that these sponges as well as other invertebrates contribute the majority of existing known marine metabolites, the protection of these organisms has become increasingly important [6]. Therefore, the optimization of methodologies to cultivate these marine organisms (or their cells responsible for generating bioactive metabolites) is crucial to produce sufficient quantities of such metabolites in order to sustain future therapeutic applications [6].

### 1.2. Deep-Sea Marine Resources off the Western Coast of Florida and Purpose of this Study

A large area of the U.S. Exclusive Economic Zone (EEZ) which harbors deep-sea marine organisms has not been extensively explored. However, as per the National Oceanic and Atmospheric Administration (NOAA) Fisheries, a multi-year collaborative study (Southeast Deep Coral Initiative (SEDCI), 2016–2019) with NOAA involving federal, academic, and local partners was launched to explore and characterize coral and sponge ecosystems within the U.S. Caribbean, U.S. South Atlantic, and the Gulf of Mexico. This study involved a large array of expeditions to survey these above-described regions and identify species of deep-sea corals and sponges.

The foremost focus of this review is to bring further attention to deep-sea corals and sponges inhabiting a specific segment of the EEZ, specifically off the western coast of Florida, using an NOAA data mining approach. Secondly, our review serves to uncover the diverse array of pharmacologically active compounds isolated from corals and sponges in the published literature, in an effort to detail their locality and the depth at which they were collected. Thirdly, this study establishes a foundation with respect to marine metabolites’ intracellular mechanism of action in mammalian cells for future therapeutic considerations. Specifically, this research inquiry evolved around mammalian cellular events, such as cytoskeletal, protein trafficking, and protein signaling processes that bioactive compounds may target. Finally, a survey of current technological advances for sustainable bioactive compound production is presented to resolve the supply problem towards the goal of minimizing the exploitation of local ecosystems. Our research serves to highlight the current gaps in knowledge and help support future research to continue investigating promising bioactive agents off the western coast of Florida.

## 2. Methods

### 2.1. Data Mining from the National Oceanic and Atmospheric Administration (NOAA) Portal

Data mining from the NOAA Deep-Sea Coral and Sponge Map Portal (www.ncei.noaa.gov/maps/deep-sea-corals/mapSites.htm (accessed on 26 May 2023 to 8 June 2023)) [8] was performed using two methods: (a) standard data download using the standard function and the map extent (spanning a 420 km by 210 km area); and (b) manual data curation from four quadrants off a defined region of the Florida western coast (spanning a 240 km by 420 km area). Map extents were captured as images. Data sets were collected into Excel files and included the scientific name, vernacular name, taxonomic classification, observation date, sea depth, latitude, longitude, and location. Data analyses were performed using Excel (2021) and the Prism GraphPad software (version 4.1.2).

### 2.2. WoRMS Database and World Porifera Database

The World Register of Marine Species (WoRMS, www.marinespecies.org/index.php (accessed on 1 September 2023 to 10 November 2023)) [9] and the World Porifera Database (www.marinespecies.org/porifera (accessed on 1 November 2023 to 10 November 2023)) [10] were accessed to obtain valid taxonomic classification of the coral and sponge data entries, identified through NOAA.

### 2.3. PubMed Literature Survey for Intracellular Mechanisms of Action

To establish the current knowledge base and gaps in knowledge regarding the mechanisms of action of bioactive compounds from corals and sponges, PubMed was exclusively utilized to systematically search through specific terms relevant to cytoskeletal elements, protein trafficking, and signaling cascades. These included the following: (a) sea coral (33, relevant article hits), (b) soft coral (104), (c) stony coral (0), (d) cup coral (0), (e) gorgonian coral (8), (f) branching coral (8), (g) black coral (0), (h) lace coral (0), (i) sea sponge (54), (j) demosponge (1), (k) calcareous sponge (0), and (l) glass sponge (0). Although the searches using the scientific names from the NOAA coral entries did not identify relevant articles, a total of 138 articles were identified for NOAA sponge entries relevant to Demospongiae, of which 78 articles were deemed scientifically relevant (and non-overlapping with the above-described search terms) for further analyses. These specific PubMed search findings are found in Supplementary Files S1–S3 for sponges (general and vernacular), corals (general and vernacular), and scientific names (sponges and corals), respectively. Full-length primary research articles were reviewed comprehensively to determine their relevancy and extract information pertinent to the topic of our investigation.

### 2.4. PubMed Literature Survey for Technological Advances Supporting the Drug Discovery Pipeline

To decipher the current state of technological developments for sustainable methods of bioactive compound production, an extensive search using keywords such as “coral cultivation methods” and “soft coral aquaculture” was performed using PubMed, resulting in 37 relevant articles for corals. Meanwhile, search terms for sponges included “in situ aquaculture”, “in vitro aquaculture”, and “cell line establishment” and uncovered 51 relevant articles. Please refer to Supplementary File S4 and Supplementary File S5 for PubMed search results on technological advances for sponges and corals, respectively.

## 3. Off the Western Coast of Florida—NOAA Analyses

### 3.1. NOAA—Standard Data Download

**Figure 1:**
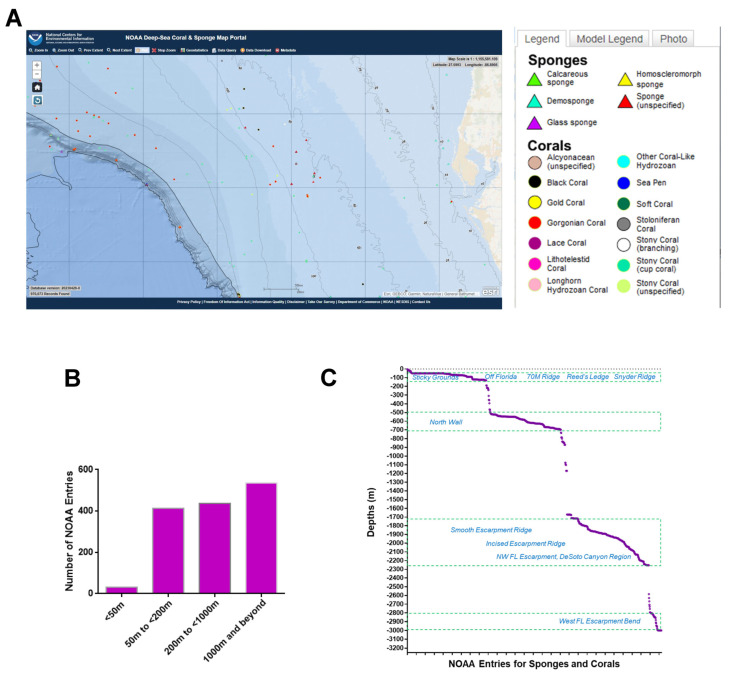
NOAA standard data mining. (**A**) Sponges and corals identified within a region (spanning a 420 km by 210 km area) off the western coast of Florida obtained using data download method from the NOAA portal. The captured map extent displays the Global Ocean and Land Terrain (GEBCO) grid contours at the indicated depths. Scale bar: 20 miles. (**B**) Number of NOAA entries representing both corals and sponges from Panel A at various depths (in meters). (**C**) Spread of NOAA entries for both corals and sponges across various depths (in meters) and ridges/escarpments.

**Figure 2:**
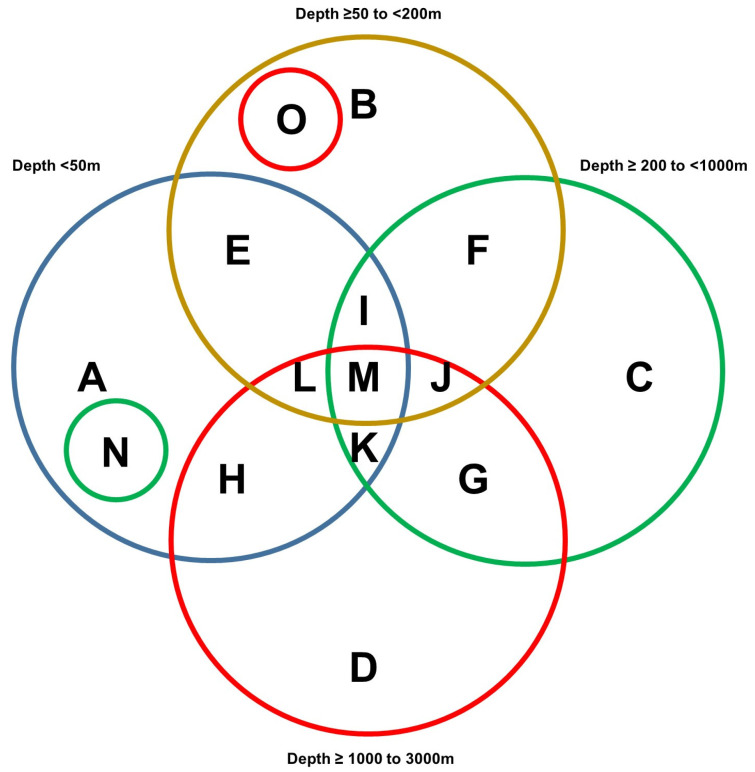
Venn diagram representation of NOAA standard data mining results. The categories are represented as follows: category A (<50 m); category B (≥50 to <200 m); category C (≥200 to <1000 m); and category D (≥1000 to 3000 m). Categories E to G represent corals and sponges collected at two different sea depths whereas categories I to L represent corals and sponges collected at three different sea depths. Category M represents sponges collected from all four sea depths whereas marine sponges and corals identified in categories O and N were uniquely found at those specific sea depths.

A region spanning a 420 km by 210 km area off the western coast of Florida (Figure 1A) was utilized as the map extent and analyzed via the standard data download function in the NOAA portal (Supplementary File S6). The data were grouped according to various sea depths as follows: (Category A) <50 m, (Category B) 50 m to <200 m, (Category C) 200 m to <1000 m, and (Category D) 1000 m to <3000 m. Categories B, C, and D represented deep-sea species (with a sea depth of more than 50 m), while category A represented shallow-sea species. As shown in Figure 1B, the analyses uncovered that 2% of the NOAA’s coral and sponge entries were found at depths of <50 m, 29% at ≥50 to <200 m, 31% at ≥200 to <1000 m, and 38% at ≥1000 to 3000 m. In Figure 1C, the NOAA entries (for both corals and sponges) are displayed at specific sea depths in association with various ridges and escarpments. The NOAA entries were subsequently analyzed for overlap amongst the various depths, as shown in Figure 2 as a Venn diagram and in Table 1. This analysis uncovered a preponderance of Porifera sponges at all sea depths, notably at ≥50 to <200 m.

### 3.2. NOAA—Manual Data Curation

**Figure 3:**
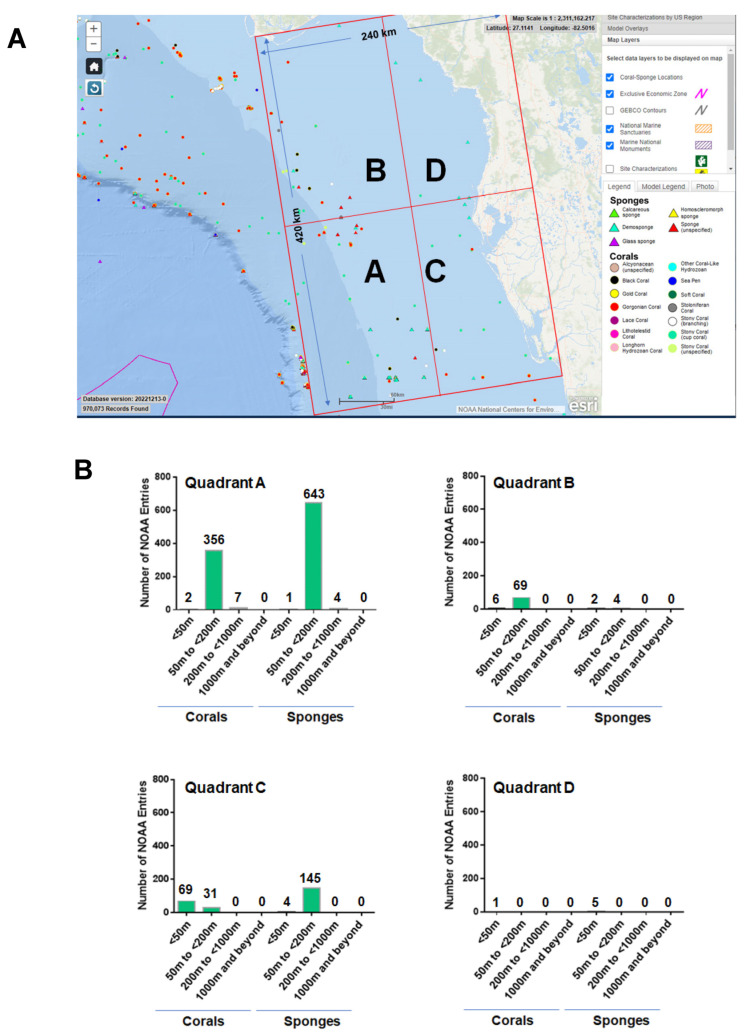
NOAA manual data mining. (**A**) Sponges and corals identified within a region (spanning a 240 km by 420 km area) off the western coast of Florida obtained manually from the NOAA portal. The captured map extent displays sponges and corals identified within four quadrants (A, B, C, and D). Scale bar: >30 miles. (**B**) Bar graph representation of the total number of NOAA entries of corals and sponges identified at varying depths within each of the four quadrants.

A region spanning a 240 km by 420 km area (separated into four quadrants, as shown in Figure 3A) off the western coast of Florida was utilized as the map extent and analyzed via manual data curation through the NOAA portal. Our manual data curation for quadrant A is found in Supplementary File S7, quadrant B is found in Supplementary File S8, quadrant C is found in Supplementary File S9, and quadrant D is found in Supplementary File S10. The NOAA entries for corals and sponges were separated and are graphically displayed in Figure 3B for each quadrant. The number of coral and sponge NOAA entries were variable between the four quadrants analyzed. It is noted that there are ~1.8-fold more sponges than corals in quadrant A at sea depths of ≥50 to <200 m, supporting the standard data download analyses. Furthermore, the number of NOAA entries representing corals were more numerous at depths of <50 m while the number of NOAA entries representing sponges were more numerous at sea depths of ≥50 to <200 in quadrant C.

### 3.3. NOAA—Taxonomic Analyses

**Figure 4:**
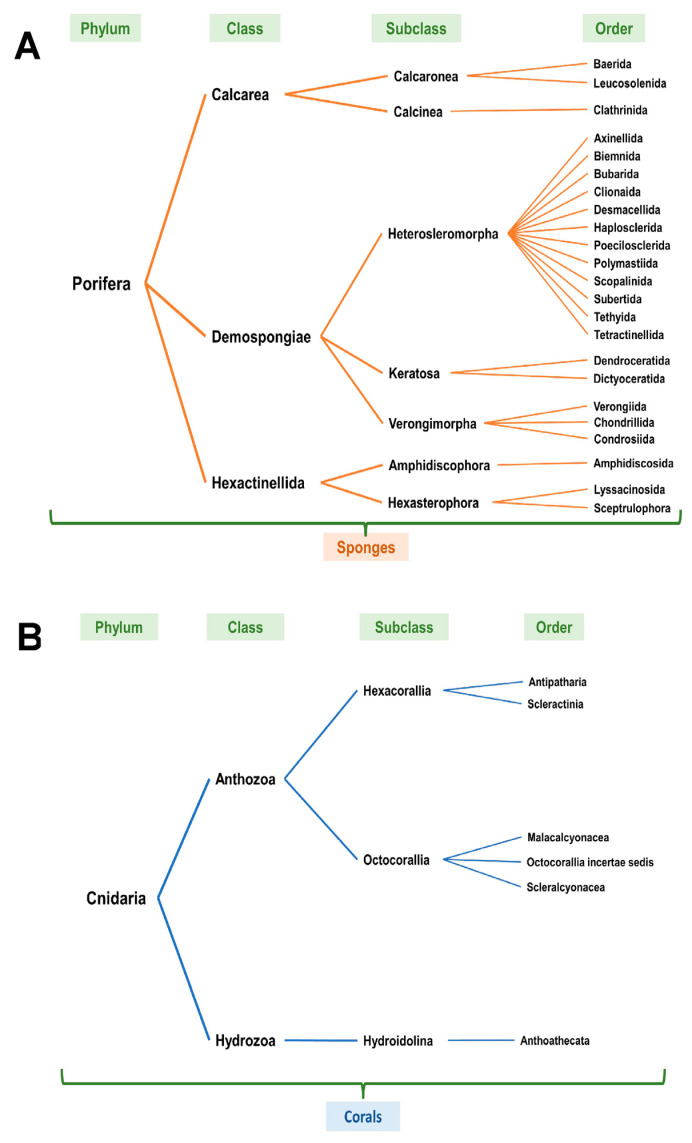
Taxonomic classification of identified corals and sponges off the western coast of Florida. (**A**) In reference to Table 1/Figure 2, classification of corals according to phylum, class, subclass, and order. Refer to Supplementary File S11 (“Corals” worksheet). (**B**) In reference to Table 1/Figure 2, classification of sponges according to phylum, class, subclass, and order. Refer to Supplementary File S11 (“Sponges” worksheet).

Upon review of the NOAA entries (based on Table 1, at all sea depths from the standard data download analyses), it was noted that the scientific name classification (taxonomic classification) was variable with only a subset identifying the specific coral or sponge species. Furthermore, each NOAA scientific entry was reviewed using the WoRMS [9] as well as the World Porifera databases [10] and revised to the current accepted scientific naming classification system (Supplementary File S11). The entries annotated in red (unaccepted) were revised to the current accepted taxonomic classification system in blue (accepted). Figure 4A,B schematically present the NOAA entries according to phylum, class, subclass, and order for corals and sponges, respectively.

## 4. Intracellular Mechanisms of Action of Bioactive Compounds

Our literature analyses using the defined search term approach (refer to Supplementary Files S1–S3) did not uncover corals or sponges from our region off the western coast of Florida (refer to Supplementary Files S12 and S13). However, it is expected that the coral and sponge species utilized in these published research studies are applicable to those in the Florida region due to their similar taxonomic classification. Furthermore, since these species are from the same class/subclass, it is expected that similar metabolites may exist for the organisms found off the western coast of Florida. The data presented in Supplementary File S12 (search term: sea coral, vernacular coral names) and Supplementary File S13 (search term: sea sponge and Demospongiae) include the time and location of the marine invertebrate collection, scientific name of the coral and sponge investigated, extract prepared, metabolite or compound synthesized, and intracellular pathway examined. The taxonomic classification using the WoRMS database [9] for each coral and sponge species can also be found in Supplementary File S12 and Supplementary File S13, respectively. It is to be noted that, with the information provided in these studies, the majority of marine corals and sponges were collected from shallow water, not deep-sea water (with only a few exceptions). The chemical structures of the bioactive compounds derived from both the corals and sponges that were identified from our searches described in Section 2.3 were obtained from the MarinLit database (https://marinlit.rsc.org (accessed on 13 November 2023 through the 18 November 2023)). The bioactive compounds derived from the corals are found in Supplementary File S14 and those from sponges are found in Supplementary File S15.

For the purpose of this review, we focused on the Anthozoa class for corals and on Demosponges for sponges. However, with respect to sponges, we also identified one article from our literature survey within the Calcarea sponge class, which is included in our discussion below.

### 4.1. Bioactive Compounds in Corals

#### 4.1.1. Cytoskeletal Alterations

**Figure 5:**
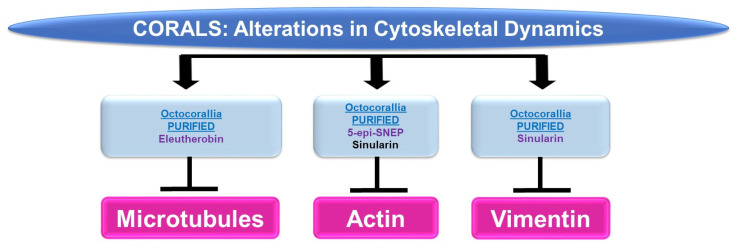
Schematic representation of the effects of coral-derived metabolites on cytoskeletal dynamics.

Please refer to Figure 5 for a schematic summary of findings relevant to cytoskeletal dynamics modulated by the bioactive compounds identified from corals.

***Microtubules:*** While marine bioactive compounds from soft corals were inhibitory to cancer cell survival, their mechanism of action via microtubules was not a common response. Although 9,11-secosterol (from *Gersemia fruticose*, a soft coral) induced cell death (10 μM, 48 h) in the K562 human leukemia cell line, it did not alter the dynamics of microtubule polymerization [11]. However, eleutherobin (from *Eleutherobia* sp., a soft coral) was capable of inducing tubulin polymerization and microtubule binding (10 μM) while inducing cytotoxicity in HCT116 colon cancer cells [12].

***Actin:*** 5-Epi-sinuleptolide (5-epi-SNEP, obtained from the soft coral *Sinularia*) reduced HeLa cervical cancer cell viability (50 μM) [13]; this functional response was associated with a reduction in actin filaments and a corresponding increase in F-actin aggregates (10 μM) as noted via phalloidin staining in confocal micrographs [13]. Sinularin (obtained from *Sinularia flexibilis*, a soft coral) reduced the cellular viability and migratory capacity of liver Sk-HEP-1 adenocarcinoma and Huh-7 hepatocellular cancer cells (10 μM) [14]; this bioactive compound also altered the morphology of F-actin filaments, which became retracted (10 μM, 24 h) [14].

***Vimentin:*** In addition to altering actin dynamics, sinularin (obtained from *Sinularia flexibilis*, a soft coral) in combination with TGFβ reduced vimentin (an intermediate filament) protein expression (1 μM, 48 h), which may have contributed to the diminished cellular migration/invasion potential of DU145 and PC3 prostate cancer cells [15].

#### 4.1.2. Protein Trafficking Alterations

**Figure 6:**
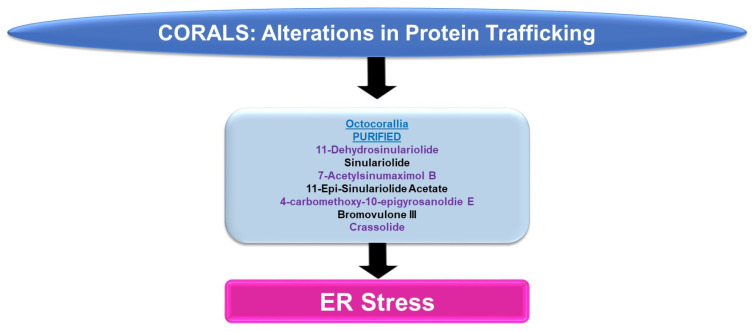
Schematic representation of the effects of coral-derived metabolites on protein trafficking.

Please refer to Figure 6 for a schematic summary of findings relevant to protein trafficking alterations modulated by the bioactive compounds identified from corals.

***ER Stress:*** An array of bioactive compounds from the genus *Sinularia* have been reported to modulate ER stress responses. This includes 11-dehydrosinulariolide (from the soft coral *Sinularia leptoclados*), which upregulates stress response pathway markers (PERK, p-PERK, CHOP, and GRP78, amongst others, at 3 μg/mL for 24 h) in oral squamous cell carcinoma cell lines [16]. The targeting of the ER stress pathway with salubrinal (Sal, an ER stress inhibitor) was shown to partially antagonize the apoptotic response (and the induction of the ER stress marker, CHOP) induced by 11-dehydrosinulariolide [16]. In A2058 melanoma cells, 11-dehydrosinulariolide also induced ER stress markers including ATF4 and CHOP (4 μg/mL for 24 h) [17]. The coincident reduction in cellular viability could also be reversed in this cell model via pre-treatment with the ER stress inhibitor salubrinal [17]. Sinulariolide (from the soft coral *Sinularia flexibilis*) induced the ER chaperones (GRP78 and GRP94) as well as p-PERK, ATF4, and CHOP (2–10 μg/mL) in hepatocellular carcinoma cell lines [18]. The induction of apoptosis coincided with ER stress and could also be partially antagonized using salubrinal [18]. 7-Acetylsinumaximol B (7-AB, from the cultured soft coral *Sinularia sandensis*) also induced ER stress characterized by elevated levels of p-PERK, ATF6, AT4, and CHOP; these alterations were accompanied by the inhibition of cell growth in the NCI-N87 gastric cancer cell line (4–32 μM) [19]. 11-Epi-sinulariolide acetate (11-epi-SA, from the cultured soft coral *Sinularia flexibilis*) induced ER stress markers (i.e., GRP78, ATF6, ATF4, and CHOP) along with the apoptotic response (9 μg/mL for 24 h), which could be partially antagonized by salubrinal or a JNK inhibitor (SP600125) [20]. Likewise, 4-carbomethoxy-10-epigyrosanoldie E (from the soft coral *Sinularia sandensis*) not only reduced the colony formation ability of Ca9-22 and Cal-27 oral cancer cells (10–20 μM) but additionally induced ER stress response markers, including GRP78, ATF4, and CHOP [21]. The ER stress inhibitor (salubrinal) additionally elicited potency in reversing the apoptotic response induced by this metabolite [21].

Bromovulone III (from the soft coral *Clavularia viridis*) induced apoptosis in liver Hep3B hepatoma cells, which occurred along with the induction of ER stress [22]. The induction of ER stress was characterized by elevated levels of CHOP/GADD153 (3 μM, 48 h) and the dilation of the ER was noted via transmission electron microscopy [22]. Crassolide (from the soft coral *Lobophytum crissum*) reduced the cellular viability of lung cancer cell lines (H460 and H1299 with IC_50_ values of 10.2 and 19.3 μM, respectively) [23]. This was accompanied by the induction of ER stress markers, including p-PERK, ATF4, and CHOP [23]. The inhibition of ER stress via 4-PBA (4-phenylbutyric acid) was able to recover lung cancer cell viability [23].

#### 4.1.3. Cell Signaling Alterations

**Figure 7:**
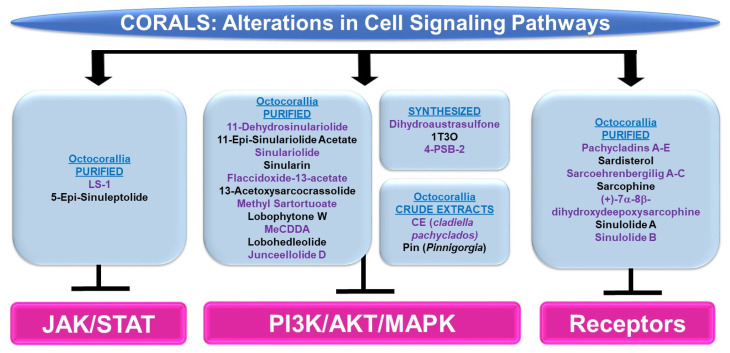
Schematic representation of the effects of coral-derived metabolites on signaling cascades.

Please refer to Figure 7 for a schematic summary of findings relevant to cell signaling alterations modulated by the bioactive compounds identified from corals.

***PI3K/AKT and/or MAPK:*** Bioactive compounds from the genus *Sinularia* have also been reported to modulate these key signaling cascades. 11-dehydrosinulariolide (from the soft coral *Sinularia flexibilis*) was tested in the neurodegeneration model, namely 6-hydroxydopamine (6-OHDA)-treated SH-SY5Y neuroblastoma cells [24]. It was noted that the agent (1 nM–1 μM) induced protection against 6-OHDA-mediated cell death, which coincided with the recovery of p-AKT and p-ERK protein levels [24]. This neuroprotection could also be inhibited using LY294002, a PI3K/AKT inhibitor [24]. 11-dehydrosinulariolide (from cultured *Sinularia flexibilis*) not only inhibited the proliferative capacity of small cell lung cancer cells (i.e., H1688, 25–50 μM, 24–48 h) but also reduced p-AKT levels along with a corresponding increase in PTEN protein (25 μM, 12–24 h) [25]. The overexpression of AKT restored the cellular viability in response to the compound (50 μM, 24 h) [25]. Another compound isolated from *Sinularia flexibilis*, 11-epi-sinulariolide acetate (11-epi-SA, 8 μM), inhibited the migration and invasion of HA22T hepatocellular carcinoma cells coinciding with reduced p-ERK1/2 and p-p38 protein levels along with reduced p-AKT, p-PI3K, and p-mTOR expression [26]. Sinulariolide (from the soft coral *Sinularia flexibilis*) not only inhibited the migratory and invasion capacity (8 μg/mL, 24–48 h) of HA22T hepatocellular carcinoma cells, but additionally reduced the protein levels of p-ERK, p-JNK, p-p38, p-AKT, and p-mTOR [27]. This agent also reduced the cellular viability and migration of TSGH-8301 human bladder cancer cells (10 μM, 24 h), which coincided with a reduction in p-PI3K, p-AKT, and p-mTOR (5–10 μM) [28]. Likewise, sinulariolide (from the cultured soft coral *Sinularia flexibilis*) reduced the cellular viability (10–14 μM, 24 h) and migratory capacity (4–10 μM, 24 h) of AGS and NCI-N87 gastric cancer cells [29]. These responses coincided with the reduced protein expression of p-PI3K, p-AKT, p-mTOR, p-JNK, p-p38, and p-ERK (10 μM) [29]. Sinularin (isolated from the cultured soft coral *Sinularia flexibilis*) not only induced an apoptotic response (20–80 μM, 24 h) in renal 786-O and ACHN cancer cell lines, but this was accompanied by reductions in the expression of p-p85-PI3K, p-AKT, and p-mTOR, while p-JNK, p-ERK, and p-p38 protein levels were elevated (via Western analyses) [30]. Furthermore, the targeting of MAPK with SB203580 (p38 inhibitor) reduced the apoptotic response [30]. The effects of sinularin were also investigated in gastric cancer cells (AGS and NCI-N87) and found to inhibit cell growth (with apoptotic features) as well as migration (18 μM, 24 h) [31]. These functional changes were associated with the reduced protein expression of p-PI3K, p-AKT, and p-mTOR (3–18 μM) [31].

Flaccidoxide-13-acetate (from the cultured soft coral *Sinularia gibberosa*) induced an apoptotic and ER stress response in RT4 and T24 bladder cancer cells, which was associated with the reduced phosphorylation of PI3K and AKT in addition to p-ERK (via Western analyses, 5–20 μM) [32]. In contrast, p-p38 and p-JNK were elevated upon cellular treatment with this bioactive compound, for which inhibitors targeting these pathways (SB203580 and SP600125, respectively) could partially restore cellular viability [32]. Also, flaccidoxide-13-acetate reduced the cellular viability (8 μM, 24 h) and the migration/invasion (4–8 μM, 24 h) of HA22T and HepG2 hepatocellular carcinoma cells [33]; these cellular responses were accompanied by reduced protein levels of p-PI3K, p-AKT, and p-mTOR [33]. Isolated from the soft coral *Cladiella kashmani*, flaccidoxide-13-acetate inhibited the cell migration and invasion of T24 and RT4 bladder cancer cells (1–10 μM), which were associated with a reduction in p-PI3K, p-AKT, and p-mTOR proteins (2.5–10 μM) [34].

13-Acetoxysarcocrassolide (13-AC, from the cultured soft coral *Sarcophyton crassocaule*) induced an apoptotic response in AGS gastric cancer cells, which was associated with reduced p-AKT, p-PI3K, and p-ERK1/2 protein levels whereas p-JNK and p-p38 levels were increased [35]. Furthermore, the use of SB203580 and SP600125 inhibitors (targeting the p38 and JNK pathways, respectively) could partially recover the cellular viability induced by 13-AC [35]. 13-Acetoxysarcocrassolide also reduced cellular viability with features of apoptosis (5 μM, 24 h) in HA22T and HepG2 hepatocellular carcinoma cells, associated with reduced p-PI3K, p-AKT, p-mTOR, and p-p70S6K proteins [36]. From the same genus, the soft coral *Sarcophyton tortuosum* was utilized to isolate methyl sartortuoate, which induced growth inhibition (with apoptotic features) in LoVo and RKO colon cancer cells (10–100 μM) [37]. This compound also induced an increase in p-JNK and p-p38 proteins (10–50 μM, 24 h) [37]. Inhibitors targeting these proteins (SP600125 and SB203580, respectively) partially restored viability [37]. A chemical library (>360 metabolites) was produced for virtual screening and molecular dynamic simulations in the context of coronaviral treatments [38]. Four compounds (**363**: sarelengan B from *Sarcophyton elegans*, **340**: bislatumlide A from *Sarcophyton latum*, **347**: dioxanyalolide from *sarcophyton elegans*, **345**: desacetylnyalolide from *Sarcophyton elegans*, and **357**: lobophytone W from *Sarcophyton elegans*) elicited strong binding affinities for the coronaviral main protease (M^pro^) [38]. The most potent was compound **340**, which, via a pathway enrichment analysis, was proposed to modulate the p38 MAPK cascade [38]. The potency of this compound is thought to arise from the large array of hydrogen bonds, hydrophobic interactions, and van der Waals interactions with key residues in the M^pro^ active site (i.e., HIS41, GLY143, MET165, GLU166, and GLN189) [38].

24-Methyl-cholesta-5,24(28)-diene-3β,19-diol-7β-monoacetate (MeCDDA, purified from the cultured soft coral *Nephthea erecta*) induced an apoptotic response in H1688 small cell lung cancer cells (20–80 μM), which was accompanied by a reduction in p-AKT and p-mTOR protein expression (10–40 μM, 24 h) [39]. Lobohedleolide (from the soft coral *Lobophytum crissum*) was shown to reduce the expression of hepatitis C viral (HCV) RNA and proteins (5–40 μM, 3 days) in an Ava-engineered HCV replicon cell line in the absence of changes in cellular survival [40]. This response was accompanied by a reduction in p-JNK protein without impacting the p-ERK or p-p38 levels [40]. Junceellolide D (JD, from the gorgonian coral *Dichotella gemmacea*) downregulated RANKL (receptor activator of nuclear factor kappa-B ligand)-induced osteoclastogenesis (1–10 μM, 5 days) and reactive oxygen species (ROS) production [41]. These cellular responses in murine bone-marrow-derived monocytes/macrophages (BMMs) were also associated with reduced p-ERK, p-JNK, and p-p38 levels [41].

The synthetic agent and precursor of austrasulfone derived from the soft coral *Cladiella australis*, namely dihydroaustrasulfone, was found to inhibit platelet-derived growth factor (PDGF)-induced migration (10 μM, 16 h) and the proliferative capacity of human aortic smooth muscle cells (HASMCs) [42]. These cellular responses were associated with reduced p-AKT and p-ERK1/2 protein levels. Furthermore, the proliferative response, as measured by the cyclin D1/E protein expression (via Western analyses), was antagonized by the PD98059 inhibitor, which targets the ERK1/2 pathway [42]. In another study, a novel compound known as 1-tosylpentan-3-one (1T3O) was synthesized (based on the precursor structure to austrasulfone) and tested on SH-SY5Y neuroblastoma cells [43]. While this compound induced protection against 6-OHDA in these cells (1 μM), 1T3O also recovered the protein expression of p-AKT and p-ERK (reduced by 6-OHDA) while p-p38 was reduced (elevated by 6-OHDA) [43]. Interestingly, LY294002, a PI3K inhibitor, inhibited the neuroprotection against cell death mediated by the synthesized marine compound [43]. Although another synthesized compound (based on a precursor to austrasulfone), namely 4-(phenylsulfanyl)butan-2-one (4-PSB-2), did not markedly alter the cellular viability of THP-1 monocytic cells (up to 20 μM, 48 h), the lipopolysaccharide (LPS)-induced reduction in CC chemokine ligand (CCL-1, a Th2 related chemokine) was associated with reduced protein levels of p-p38, p-ERK, and p-JNK [44].

A crude organic ethyl acetate extract generated from the soft coral *Cladiella pachyclados* (CE, characterized by 20 compounds (sterols, terpenoids, alkaloids, eunicellin-type, and cembrane-type)) was cytotoxic towards MCF-7 and MDA-MB-231 breast cancer cells (with IC_50_ values of 24 μg/mL and 10 μg/mL, respectively) [45]. Using publicly available databases, targets were identified within the MAP kinase signaling network, with ERK1 being identified as a highly interactive protein [45]. Another crude extract (Pin) generated from the gorgonian coral *Pinnigorgia* was found to inhibit cellular viability (2–6 μg/mL, 24 h) with associated apoptotic features in HSC-T7 rat hepatic stellate cells [46]. This was associated with elevated levels of p-ERK and p-JNK (via Western blotting) [46]; the use of JNK and ERK inhibitors (i.e., SP600125 and PD98059, respectively) could antagonize the reduced cellular viability induced by Pin (4 μg/mL, 24 h) [46].

***JAK/STAT:*** Altogether, there were few articles identified using our search terms for bioactive compounds derived from corals eliciting alterations in the JAK/STAT signaling cascade. (1S,2S,3E,7E,11E)-3,7,11,15-cembratetraen-17,2-olide (LS-1, from the soft coral *Lobophytum*) elicited an inhibitory effect in HT-29 colon cancer cells associated with apoptotic features (0.1–10 μM, 48 h) [47]. This response was accompanied by a reduction in both p-STAT3 and total STAT3 proteins [47]. These changes also occurred with protein changes in AKT and MAPK pathway mediators [47]. 5-Epi-sinuleptolide (from the soft coral *Sinularia leptoclados*) was cytotoxic towards BxPC-3 and PANC-1 pancreatic cancer cells (10–50 μM), with apoptotic features [48]. The bioactive compound also attenuated the protein level of p-JAK2 and p-STAT3 in addition to p-AKT and p-ERK1/2 in these cells [48].

***Cell Surface Signaling Receptors:*** Pachycladins A–E (compounds **1**–**5**, from the soft coral *Cladiella pachyclados*) were tested in a series of breast cancer cell lines [49]. Specifically, pachycladin A (compound **1**) reduced the growth of MDA-MB-231 breast cancer cells (EC_50_ of 1.6 μM) [49]; this was accompanied by the inhibition of EGFR kinase activity (10 μM) as well as p-EGFR protein levels (0.5–4 μM) [49]. Molecular modeling studies uncovered that, using the EGFR crystal structure (Protein Data Bank (PDB): 2ITW and 4WKQ), the compound fit well into the ATP binding pocket of the kinase with hydrogen bonds with MET793 and PRO794, which exist within the hinge region of the kinase domain [49]. Pachycladin A also mediated contact with a hydrophobic region with side chains of VAL726, ALA743, LYS745, GLN791, and LEU792 [49]. A series of metabolites were purified from the soft coral *Sarcophyton ehrenbergi* (namely, sarcoehrenbergilids A-C, sarcophine, (+)-7α-8β-dihydroxydeepoxysarcophine, sinulolide A, sinulolide B, and sardisterol) [50]. While all these compounds elicited anti-proliferative activity towards an array of cancer cell lines, sardisterol elicited the most potent response in lung A549 cancer cells [50]. Molecular docking simulations were performed using AutoDock to uncover the binding capacity of sardisterol to EGFR [50]; this uncovered two stabilizing hydrogen bonds with the active site along with van der Waals and hydrophobic interactions involving LEU865, LEU694, and VAL702 [50].

### 4.2. Bioactive Compounds in Sponges

#### 4.2.1. Cytoskeletal Alterations

**Figure 8:**
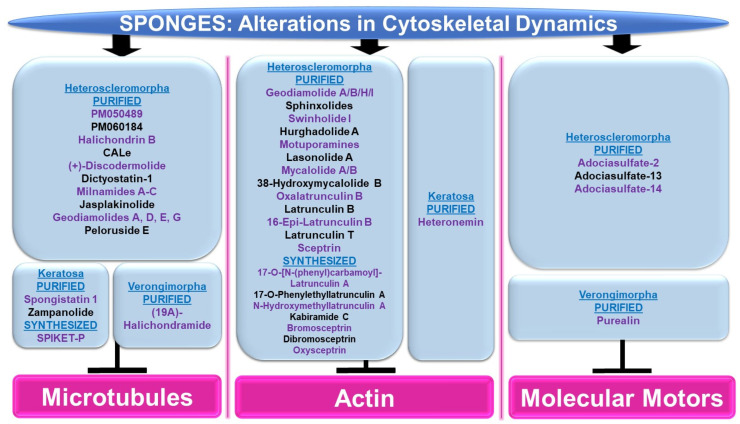
Schematic representation of the effects of sponge-derived metabolites on cytoskeletal dynamics.

Please refer to Figure 8 for a schematic summary of findings that are relevant to alterations in cytoskeletal dynamics modulated by the bioactive compounds identified from sponges.

***Microtubules:*** Although the majority of bioactive compounds from sponges that were identified with effects on microtubules were within the subclass of Heteroscleromorpha, a smaller subset of sponge-derived compounds were also identified within the subclass of Keratosa and Verongimorpha. All these studies focused on anticancer therapies.

*Subclass of Heteroscleromorpha:* PM050489 and PM060184 (obtained from *Lithoplocamia lithistioides*) induced mitotic phase arrest in lung A549 cancer and breast MDA-MB-231 cancer cells [51]. This cellular response to these compounds was suggested to be mediated by the disruption of microtubules by binding to the β-tubulin (+)-end (at ASN100) with high affinity [52]. Halichondrin B (obtained from *Axinella* sp.) induced a potent cytotoxic response associated with mitotic arrest in murine leukemic cells (with IC_50_ values of 0.3 nM) [53]. This response was associated with the inhibition of vinblastine’s interaction with tubulin in a non-competitive manner [53]. While *N*-formyl-7-amino-11-cycloamphilectene (CALe, obtained from *Axinella* sp.) did not induce a cytotoxic response in HeLa cervical cancer cells, the agent was found to inhibit the colcemid-induced polymerization of microtubules (100 μM, 2 h) [54]. (+)-Discodermolide (obtained from *Discodermia dissoluta*) induced mitotic arrest in MCF-7 breast cancer cells (with IC_50_ values of 2.4 nM) [55]. Via immunofluorescence staining with the β-tubulin antibody, this compound (1 μM) caused microtubule rearrangement characterized by filamentous bundling associated with retraction around the nuclear compartment [55]. Dictyostatin-1 (obtained from the sponge family *Corallistidae*) elicited a potent cytotoxic response in lung A549 cancer cells (with IC_50_ values of 0.95 nM) [56]. This was associated with microtubule condensation (10–1000 nM, as noted via immunofluorescence staining with the α-tubulin antibody) [56]. Bioactive compounds (milnamides A–C, jasplakinolide, and geodiamolides A/D/E/G) derived from *Auletta* sp. were found to disrupt cytoskeletal dynamics along with cellular cytotoxicity in MDA-MB-435 melanoma cells [57]. Specifically, via immunofluorescence studies, while milnamide B (0.1 μg/mL) and milnamide C (10 μg/mL) caused microtubule disruptions, geodiamolide E (1 μg/mL) caused actin filament disorganization [57]. Peloruside E (obtained from *Mycale hentscheli*) inhibited the cellular growth of HL-60 cells (with IC_50_ values of 90 nM) [58]. Furthermore, the ability of the novel compound (2.8 nM) to induce tubulin polymerization was slower than that for peloruside A [58].

*Subclass of Keratosa:* SPIKET-P (synthesized based on spongistatin 1 derived from the sponge genus *Hytrios*) induced a potent cell death response in BT-20 breast cancer cells that was associated with microtubule disorganization (10 nM for 24 h, assessed via immunofluorescence staining with α-tubulin) [59]. Spongistatin 1 (obtained from *Hytrios erecta*) caused the disruption of microtubular networks in the fungus *Cryptococcus neoformans*, as determined by immunofluorescence α-tubulin antibody staining [60]. Zampanolide (obtained from *Cacospongia mycofijiensis*) induced cell death in promyelocytic HL-60 cells (2–10 nM, 24–48 h), associated with G2/M phase arrest [61]. Furthermore, the compound induced microtubule bundling and aster formation (10 nM, 12 h) [61].

*Subclass of Verongimorpha:* (19Z)-Halichondramide (obtained from *Chondrosia corticate*) induced a potent inhibitory effect on cellular proliferation across a series of cancer cell lines including A549 lung cancer cells (with IC_50_ values of 0.024 μM) [62]. This was associated with the inhibition of β-tubulin polymerization via immunofluorescence staining (25–50 μM, 24 h) and G2/M phase arrest [62].

***Actin:*** The majority of sponge-derived bioactive compounds identified with effects on actin were within the subclass of Heteroscleromorpha with only one article identified within the subclass of Keratosa. All the studies uncovered were also categorized, like the microtubules, in the context of anticancer therapies.

*Subclass of Heteroscleromorpha:* Geodiamolides A/B/H/I (obtained from *Geodia corticostylifera*) induced F-actin disorganization and aggregation in a dose-dependent manner (50–100 ng/mL, 28 h) in T47D breast cancer cells [63]. Geodiamolide H (obtained from *Geodia corticostylifera*) caused some regions of 3D spheroid cultures of Hs578T breast cancer cells to lose actin filaments (as observed via phalloidin staining) [64]. This cellular feature may have caused the reduced cellular migratory and invasive potential in 2D cultures (10–120 nM, 24 h) [64]. Sphinxolides (four purified compounds, obtained from *Neosiphonia superstes*) caused a reduction in cellular proliferation that was associated with G2/M phase arrest (with IC_50_ values of 10–60 nM) across a series of cancer cell lines [65]. Specifically, sphinxolide B caused defects in actin polymerization in in vitro assays [65]. Two bioactive compounds, namely swinholide I and hurghadolide A (obtained from *Theonella swinhoei*), induced cytotoxicity in a series of colon cancer cell lines (with IC_50_ values of 5.6 nM and 365 nM, respectively) [66]. However, hurghadolide A was 10 times more potent in disrupting actin microfilaments, relative to swinholide I [66]. Although motuporamines (obtained from *Xestospongia exigua*) did not alter cellular proliferation in MDA-MB-231 breast cancer cells (4–8 μM), the compound did cause a morphological alteration with features of actin aggregation (as noted via rhodamine-phalloidin staining) [67]. This appeared to be associated with reduced MDA-MB-231 cell migratory capacity (5 μM, 24 h) [67]. Lasonolide A (obtained from *Forcepia* sp.) not only reduced cell numbers across an array of pancreatic and breast cancer cell lines (with a TC_50_ range of 25 nM to 90 nM), but also induced cellular contraction associated with a reduction in actin filaments (via phalloidin staining) in Panc-1 pancreatic cancer cells [68]. A crude extract and three bioactive compounds, namely mycalolide A, mycalolide B, and 38-hydroxymycalolide B (obtained from *Mycale nullarosette*), caused a marked reduction in F-actin staining (via phalloidin) as well as actin filament depolymerization [69].

Via molecular modeling, oxalatrunculin B (obtained from *Negombata corticata*) was predicted to bind poorly to the G-actin crystal structure (1RDW) [70]. In support, in an in vitro actin polymerization assay, oxalatrunculin B was less potent compared to latrunculin (0.15–15 μM compared to 2.56 μM) [70]. Other naturally derived latrunculins from this sponge include latrunculin B, 16-epi-latrunculin B, and latrunculin T [71]. Molecular docking analyses identified that the structural differences (e.g., epimerization and macrolide ring opening) amongst these compounds mediates their effectiveness in binding to G-actin [71]. Latrunculin B elicited the most cytotoxic response in HCT116 colon cancer cells (with IC_50_ values of 18.62 μM) [71]. A series of semi-synthetic analogues based on the structure of latrunculin A (which is well established to interact with G-actin in a reversible manner to hinder actin polymerization with hydrogen bond contact points at TYR69, THR186, ARG210, and ASP157 [72]) were tested to determine their inhibitory potency on actin polymerization [73]. While latrunculin A was potently cytotoxic (500 nM), 17-O-[N-(phenyl)carbamoyl]-latrunculin A was far more cytotoxic (100 nM), suggesting that the chemical modifications on latrunculin were critical to mediating its potency [73]. Specifically, this involved a hydrogen bond with ARG210 of actin, as determined via molecular docking studies [73]. Other semi-synthetic analogs, namely 17-*O*-phenylethyllatrunculin A and N-hydroxymethyllatrunculin A, also elicited more potency compared to latrunculin A (at 100 nM and 1 μM) in an in vitro actin polymerization assay [74]. Further, these compounds were cytotoxic (0.1–50 μM) in the MCF-7 and MDA-MB-231 breast cancer cell lines [74]. Via molecular docking, 17-O-phenylethyllatrunculin A showed an optimal electrostatic interaction with ARG210 along with strong hydrogen bonds with TYR69, ASP157, THR186, and GLU214 while N-hydroxymethyllatrunculin A showed strong hydrogen bond formation with ASP157 [74].

Semi-synthetic analogues of kabiramide C (obtained from *Pachastrissa nux*), namely 7-azidokabiramide C, 7-[4-*N*-(9*H*-fluoren-9-ylmethoxycarbonyl)aminomethyl-1,2,3-triazol-1-yl]kabiramide C, and 7-(-aminomethyl-1*H*-1,2,3-triazol-1-yl)kabiramide C) were demonstrated to bind tightly to G-actin (0.17 μM to 1.96 μM) [75]. In addition, the latter product induced cytokinesis defects in the HeLa cervical cancer cell line (10–100 nM, 16 h) [75]. The cellular response to purified sceptrin (obtained from *Agelas nakamurai*) as well as its synthetic form and derivatives (bromosceptrin, dibromosceptrin, and oxysceptrin) were tested across a series of cancer cell lines [76]. While spectrin did not modulate cellular proliferation (40 μM, 24 h), it (and its derivatives) inhibited cellular migration [76]. Spectrin also induced lamellipodia formation in HeLa cervical cancer cells as well as inhibited clot retraction, which appeared to be mediated by direct binding to monomeric actin, as determined by isothermal titration calorimetry studies [76].

*Subclass of Keratosa:* Heteronemin (obtained from *Hippospongia* sp.) elicited the most potent cytotoxic response in leukemic cells (with an IC_50_ value of 0.1 (MOLT4)—0.41 mg/mL, 28 h) that was associated with apoptosis [77]. Using MOLT4 cells, the bioactive compound increased talin and p-talin protein levels (a cytoskeletal protein located at cell–cell contacts) as well as altered actin extensions (via immunofluorescence staining) [77].

***Molecular Motors:*** The majority of sponge-derived metabolites identified with effects on molecular motors such as kinesin and dynein were within the subclass of Heteroscleomorpha with one article identified in the subclass of Verongimorpha.

*Subclass of Heteroscleromorpha:* An extract (derived from *Haliclona* sp.) was found to inhibit microtubule attachment to kinesins in the microtubule gliding motility assay, which coincided with the complete inhibition of kinesin ATPase activity [78]. Furthermore, adociasulfate-2 (AS-2) was purified as the active component that mediated these activities (with IC_50_ values at low μM values) [78]; this compound (35 μM) also disrupted kinesin to microtubule interactions, as determined in a co-sedimentation assay [78]. In an independent study, AS-2 was shown to inhibit the ATPase activity of CENO-E and Eg5 kinesins, either in the presence or absence of microtubules [79]. AS-2 also promoted the release of ADP from the CENP-E [79]. Adociasulfate-13 and adociasulfate-14 (obtained from *Cladocroce aculeata*) were both shown to inhibit kinesin binding to microtubule-bound beads in the absence of compound aggregation [80].

*Subclass of Verongimorpha:* A synthetic form of purealin (obtained from *Psammaplysilla purea*) was demonstrated to inhibit the ATPase activity of a recombinant form of the dynein motor protein in a dynein motor domain ATPase inhibition study (50 μM) [81].

#### 4.2.2. Protein Trafficking Alterations

**Figure 9:**
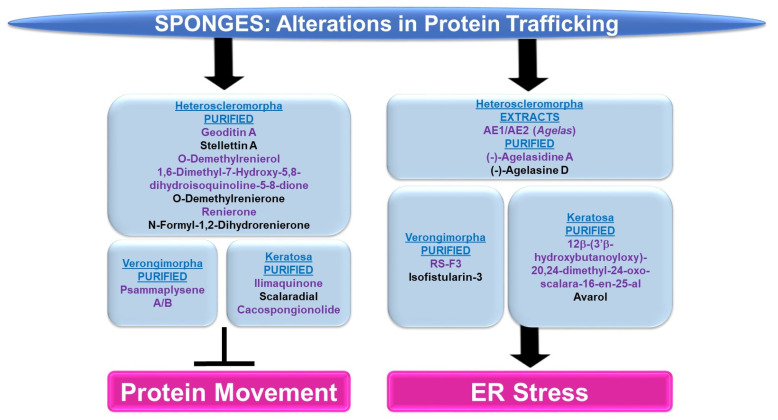
Schematic representation of the effects of sponge-derived metabolites on protein trafficking.

Please refer to Figure 9 for a schematic summary of findings relevant to alterations in protein trafficking modulated by the bioactive compounds identified from sponges.

***Protein Movement:*** The majority of sponge-derived bioactive compounds identified with effects on protein movement were within the subclass of Heteroscleromorpha and Keratosa with a smaller subset identified within the subclass of Verongimorpha.

*Subclass of Heteroscleromorpha:* Geoditin A (derived from *Geodia japonica*) induced cellular cytotoxicity (with IC_50_ values of 10 μg/mL) and inhibited L-DOPA conversion (needed for melanogenesis) in B16F10 melanoma cells [82]. Tyrosinase, responsible for L-DOPA conversion, was reduced in the protein levels, misdirected to the ER and lysosomes (rather than the Golgi apparatus), and incompletely processed at the glycosylation level [82]. Stellettin A (obtained from *Geodia japonica*) was highly cytotoxic to B16F10 murine melanoma cells (with IC_50_ values of 2 μg/mL, 24 h) [83]. Similar to the effect of geoditin A, stelletin A caused tyrosinase-related protein 1 (TRP-1) to be incorrectly processed coinciding with the induction of the unfolded response pathway (UPR) [83]. Novel isoquinolinequinones (derived from *Haliclona* sp.), namely O-demethylrenierol, 1,6-dimethyl-7-hydroxy-5,8-dihydroisoquinoline-5-8-dione, O-demethylrenierone, renierone, and N-formyl-1,2-dihydrorenierone, reduced the nuclear translocation of the p65 subunit of NFκB (1–10 μM) in a co-culture of Caco-2 and THP-1 cells treated with LPS/IFN-γ in the absence of cytotoxic effects [84].

*Subclass of Verongimorpha:* Two bioactive compounds, namely psammaplysene A and psammaplysene B (purified from *Psammaplysilla* sp.), could restore tumor-suppressive activity in PTEN-deficient cells via the inhibition of FOXO1a nuclear export (5 μM and 20 μM, respectively) in the absence of alterations in CRM1 or p-AKT levels [85].

*Subclass of Keratosa:* Ilimaquinone (IQ, obtained from *Hippospongia metachromia*) led to the fragmentation of the Golgi apparatus in normal rat kidney cells [86]. Furthermore, the protein trafficking of vesicular stomatitis virus (VSV, used as a reporter) was blocked by IQ between two Golgi-enriched membrane fractions in a cell-free transport assay [86]. IQ hindered β-COP proteins and ARF (GTP-binding protein)’s interaction with the Golgi [86]. In an independent study, IQ elicited a potent cytotoxic response across an array of cancer cell lines in a dose-dependent manner (0.3–30 μM) that was associated with increased G1/sub-G1 populations [87]. These cellular responses were accompanied with Golgi fragmentation within 6 h post-treatment [87]. Furthermore, the nuclear translocation of the p65 subunit of NFκB was reduced by IQ (via immunofluorescence staining) [87]. Scalaradial (SC) and cacospongionolide (CSP) (obtained from *Cacospongia scalaris* and *Fasciospongia cavernosa*, respectively) caused a dose-dependent inhibition of the viability of cancer cells (T47D breast, A431 epidermoid, and HeLa cervical) with apoptotic features (10 μg/mL, 24 h) [88]. These cellular responses were associated with a reduction in the nuclear translocation of both the p50 and p65 subunits of NFκB (10 μg/mL, 24 h) [88].

***ER Stress:*** The sponge-derived metabolites identified with effects on ER stress were within the subclasses of Heteroscleomorpha, Verongimorpha, and Keratosa.

*Subclass of Heteroscleromorpha:* Crude extracts (AE1 and AE2, prepared from *Agelas* sponges) (5 μg/mL) sensitized cancer cell lines to irradiation [89]. This was associated with the induction of the ER stress response, characterized by elevated levels of CHOP and ATF4 [89]. (−)-Agelasidine A (derived from *Agelas nakamurai*) induced cytotoxicity in HepG2 and Hep3B liver cancer cell lines (with IC_50_ values of 129 μM and 69.9 μM, respectively), associated with apoptotic features and ER stress (elevated levels of GRP78, CHOP, ATF4, and p-PERK) [90]. The ER stress inhibitor 4-PBA (4-phenylbutyric acid) restored the cell viability and reduced apoptotic characteristics, in combination with (−)-agelasidine A [90]. (−)-Agelasine D (derived from the *Agelas* sponge) induced a potent cytotoxic response across a series of liver cancer cell lines (with a GI_50_ of 9.9 μM), which coincided with the induction of ER stress (elevated levels of p-ERK and ATF4 proteins) in Hep3B cells [91].

*Subclass of Verongimorpha:* (+)-1(R),6(S),1′(R),6′(S),11(R),17(S)-fistularin-3 (RS-F3, derived from *Suberea clavata*) reduced the cellular viability of acute myeloid leukemia cells (GI_50_ of 3.2 to 15.17 μM) [92]. This was associated with an elevated ER stress response (15 μM, 24 h) characterized by increased levels of p-PERK and CHOP protein as well as increased RNA levels of an XBP1 (X-Box binding protein 1) splice variant [92]. Isofistularin-3 (Iso-3, derived from *Aplysina aerophoba*) sensitized cells to TRAIL in RAJI and U-937 cancer cell lines (pre-treatment at 15 μM) [93]; this was associated with apoptosis and ER stress (elevated GRP78 protein as well as CHOP RNA levels) [93].

*Subclass of Keratosa:* 12β-(3′β-hydroxybutanoyloxy)-20,24-dimethyl-24-oxo-scalara-16-en-25-al (purified from *Carteriospongia (Phyllospongia)* sp.) induced cytotoxic responses in an array of leukemic and lymphoma cell lines (with IC_50_ values of 2.08 nM) such as MOLT4 cells [94]. This response was associated with ER stress, characterized by elevated levels of CHOP, GRP94, and ATF6 (0.0625 μg/mL, 9 h) [94]. Avarol (purified from *Dysidea avara*) inhibited cell viability in an array of cancer cell lines, coinciding with the induction of ER stress (elevated levels of BiP and CHOP RNA and protein) [95]. Furthermore, CHOP siRNA antagonized an avarol-mediated apoptotic response [95].

#### 4.2.3. Cell Signaling Alterations

**Figure 10:**
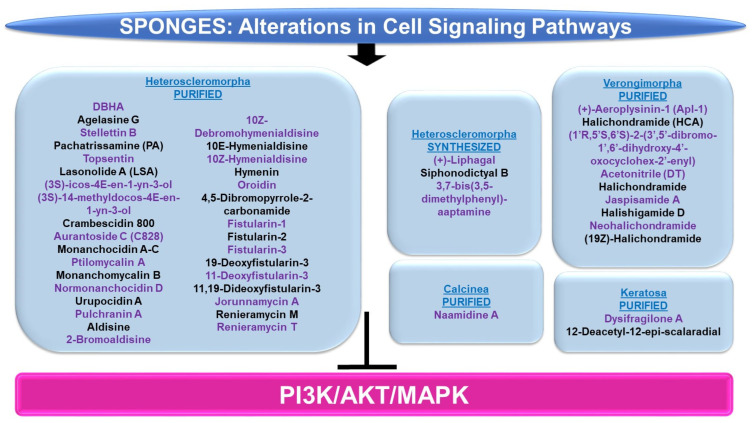
Schematic representation of the effects of sponge-derived metabolites on the PI3K/AKT and MAPK signaling networks.

**Figure 11:**
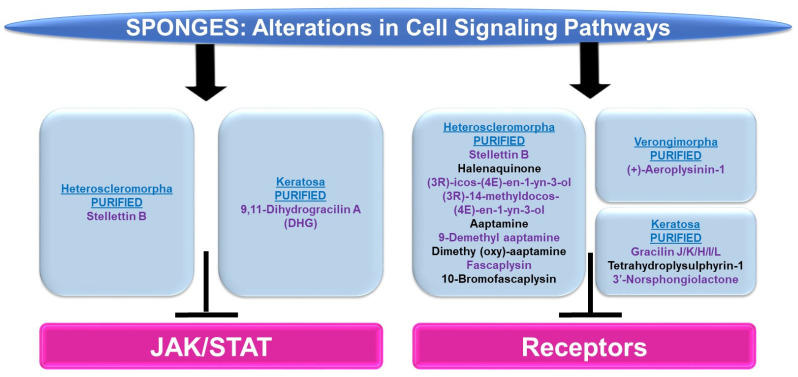
Schematic representation of the effects of sponge-derived metabolites on the JAK/STAT and cell surface receptor signaling networks.

Please refer to Figure 10 and Figure 11 for a schematic summary of findings relevant to alterations in cell signaling modulated by the bioactive compounds identified from sponges.

***PI3K/AKT and/or MAPK:*** The majority of sponge-derived metabolites identified with effects on PI3K/AKT and MAPK signaling were within the subclass of Heteroscleromorpha with a smaller subset identified within the subclasses of Verongimorpha and Keratosa.

*Subclass of Heteroscleromorpha:* 6″-Debromohamacanthin A (DBHA, derived from *Spongosorites* sp.) reduced cellular migration and tube formation of vascular endothelial growth factor (VEGF)-stimulated HUVECs (up to 10 μM) [96]; these functional responses were associated with reduced levels of p-AKT, p-PDK1, p-mTOR, and p-p70S6K [96]. Agelasine G (obtained from *Agelas nakamurai*) inhibits protein tyrosine phosphatase (PTP, a target for type II diabetes therapy) with an IC_50_ value of 15 μM [97]. This compound also increased the p-AKT levels in response to insulin stimulation in Huh-7 hepatocellular carcinoma cells (20–50 μM, 2 h) [97]. Stellettin B (purified from *Jaspis stellifera*) reduced the proliferation capacity of A549 lung cancer cells (with an IC_50_ value of 0.022 μM) with features of apoptosis [98]. This response was accompanied by reduced protein levels of p-PDK1, p-AKT, p-mTOR, and p-p70S6K, whereas no effect was noted on p-p38 or p-ERK (0.02–1 μM, 24 h) [98]. In an independent study, stellettin B also was found to potently inhibit cell growth in the SF295 glioblastoma cell line (GI_50_ of 0.03 μM), associated with apoptotic features [99]. These cellular changes were associated with reduced p-AKT protein levels (and PI3K activity, 10 μM) in the absence of alterations in p-p38 or p-ERK [99]. In a separate study, this bioactive compound was found to mediate neuroprotection against apoptosis in SH-SY5Y neuroblastoma cells treated with 6-OHDA (0.1–100 nM, 1 h pre-treatment) [100]. This was accompanied by the recovery of downregulated p-ERK and p-AKT (0.1 nM stellettin B) whereas the elevated p-p38 induced by 6-OHDA was reduced [100]. Furthermore, the targeting of the PI3K pathway using LY294002 (a PI3K inhibitor) reduced the neuroprotection mediated by stellettin B in this cell model [100]. Pachatrissamine (PA, purified from *Pachastrissa* sp.) inhibited the cellular proliferation of A375 and B16F10 melanoma cells lines (5 μg/mL, 24 h) accompanied by apoptotic features. While p-ERK was reduced by the bioactive compound, p-AKT remained unchanged (5 μg/mL, 30 min). Topsentin (purified from *Spongosorites genitrix*) was investigated in HaCaT keratinocytes stimulated with ultraviolet B (UVB) irradiation [101]. While the compound did not induce significant cellular toxicity (up to 10 μM), Topsentin inhibited COX-2 expression (RNA and protein) which was accompanied by reduced p-p38, p-ERK, and p-JNK proteins in a dose-dependent manner (2.5–10 μM) [101]. Lasonolide A (LSA, purified from *Forcepia* sp.) was found to target RAF1 via an shRNA kinase screening approach [102]. Furthermore, LSA activated RAF1 at SER338 in the HCT116, OVCAR-8, and CA46 cancer cell lines [102]. The marine metabolites (3S)-icos-4E-en-1-yn-3-ol and (3S)-14-methyldocos-4E-en-1-yn-3-ol (derived from *Cribrochalina vasculum*) induced cellular cytotoxicity in lung cancer cell lines, including U-1810, a non-small cell lung cancer cell line (NSCLC) [103]. This response was associated with a reduction in p-AKT, p-mTOR, and p-ERK1/2 in contrast to p-JNK1/2 (3 μmol/L, 4–24 h) [103]. Crambescidin 800 (C800, purified from *Monanchora viridis*) reduced cellular viability across a series of breast cancer cell lines including T11 cells (with an IC_50_ value of 0.07 μM, 72 h), associated with G2/M phase arrest [104]. These functional consequences were associated with reduced p-AKT, p-mTOR, and p-p42/44-MAPK (20 μM, 24 h) [104]. Aurantoside C (C828, purified from *Manihinea lynbeazleyae*) was cytotoxic towards triple negative breast cancer cell lines including SUM159PT (with an IC_50_ value of 0.56 μM), associated with S phase arrest [105]. These functional changes were associated with increased levels of p-AKT at low doses of C828 (0.01–1 μM) but with reduced levels at high doses (5 μM) [105]. At these C828 high doses, p-SAPK/JNK were, in contrast, increased in SUM159PT cells [105].

An array of bioactive compounds (obtained from *Monanchora pulchra*), namely monanchocidin A, monanchocidin B, monanchomycalin C, ptilomycalin A, monanchomycalin B, normonanchocidin D, urupocidin A, and pulchranin A, were tested in JB6 P+ CI41 epidermal cells which were stimulated with epidermal growth factor (EGF) [106]. The majority of these compounds antagonized the anchorage-independent colony formation (at low μM/nM doses), likely through an apoptotic response [106]. Moreover, the compounds induced p-JNK and p-ERK protein expression in JB6 P+ CI41 cells [106]. A series of bioactive compounds (purified from *Stylissa massa*), namely aldisine, 2-bromoaldisine, 10Z-debromohymenialdisine, 10E-hymenialdisine, 10Z-hymenialdisine, hymenin, oroidin, and 4,5-dibromopyrrole-2-carbonamide, were assessed for their activities towards MEK1 and Raf [107]. Specifically, 10E-hymenialdisine and 10Z-hymenialdisine were the most potent (with moderate activity elicited by 2-bromoaldisine and 10Z-debromohymenialdisine) in inhibiting MAPK (at THR202 and TYR204) as assessed via an ELISA as well as the most effective in inhibiting the cellular viability of LoVo adenocarcinoma large intestinal cells [107]. The compounds had no effect on Raf [107]. A series of fistularins (purified from *Ecionemia acervus*), namely fistularin-1, fistularin-2, fistularin-3, 19-deoxyfistularin-3, 11-deoxyfistularin-3, and 11,19-dideoxyfistularin-3, were tested (up to 20 μg/mL) in a co-culture system of THP-1 (monocytic cells) and Caco-2 (epithelial intestinal cells), treated with LPS and IFN-γ, for their ability to modulate the expression of pro-inflammatory markers, iNOS and COX-2 [108]. All of the fistularins inhibited iNOS and COX-2 protein levels in addition to cytokine (IL-1β, IL-6, and TNF-α) as measured via an ELISA [108]. These changes were accompanied by reduced p-ERK, p-p38, and p-JNK protein expression under the same culture conditions [108].

Jorunnamycin A (derived from *Xestospongia* sp.) inhibited the cellular proliferation of adherent H460 lung cancer cells (0.1–0.5 μM, 72 h) and furthermore, in a suspension culture, phosphatidylserine (PS) exposure, a marker of apoptosis, was noted (0.5 μM, 24 h) [109]. This cell death response was accompanied by reduced levels of p-AKT and p-ERK (0.05–0.5 μM, 12 h) [109]. Renieramycin M (RM, obtained from *Xestospongia* sp.) induced a 60-fold increased (with an IC_50_ value of 6 nM) cytotoxicity in MCF-7 breast cancer cells, relative to doxorubicin (with an IC_50_ value of 356 nM) [110]. This potent effect appeared to be mediated through alterations in the ErbB/PI3K/AKT pathways, as determined by microarray-based transcriptomic analyses [110]. A synthetic derivative of renieramycin T (*O*-acetyl RT) reduced cellular viability (with an IC_50_ value of 0.66 μM) in H292 non-small cell lung cancer cells, which was accompanied by apoptotic features [111]. In addition to the loss of cancer stem cell characteristics (reduced Nanog and CD44 protein), p-AKT levels were reduced with *O*-acetyl RT treatment [111].

Chemically synthesized (+)-liphagal (originally derived from *Aka coralliphaga*) and its derivatives were assayed for their ability to inhibit the enzymatic activity of PI3K-α and PI3K-γ [112]. One synthetic derivative demonstrated specificity towards the PI3K-α isoform (with IC_50_ values of 66 nM, compared to IC_50_ values of 1840 nM towards the PI3K-γ isoform) [112]. In an independent study, chemically synthesized liphagal and siphonodictyal B were cytotoxic (associated with an apoptotic response, 0.5–1 μM, 48 h) in HCT116 colon cancer cells [113]. This was associated with signaling pathway alterations characterized by increased p-p38 levels (1 μM, 24 h) [113]. Chemically modified aaptamine (purified from *Aaptos aaptos*), namely 3,7-bis(3,5-dimethylphenyl)-aaptamine (AP-51), induced apoptosis in a dose-dependent manner (1.25–5 μM) in Raji (Burkitt lymphoma) cancer cells, which was associated with a reduction in p-PI3K, p-AKT, and p-mTOR proteins [114].

*Subclass of Verongimorpha:* (+)-Aeroplysinin-1 (Apl-1, derived from *Aplysina aerophoba*) was investigated with respect to the mechanism of action of its anti-inflammatory effects in human umbilical vein endothelial cells (HUVECs) [115]. While TNF-α induced the transcript expression of CCL2, ICAM1, SELE, and IL-6, these cytokine levels were reduced by Apl-1 [115]. From a mechanistic signaling perspective, Apl-1 was found to reduce p-AKT protein levels induced by TNF-α [115]. Halichondramide (HCA, obtained from *Chondrosia corticata*) inhibited PC3 prostate cancer cell proliferation (with IC_50_ values of 0.81 μM, 72 h) as well as wound healing (50–800 nM) [116]. This bioactive agent also reduced the levels of the p85 and p110 subunits of PI3K RNA (100 nM, 24 h) and protein (50–800 nM, 24 h) [116]. (1′R,5′S,6′S)-2-(3′,5′-dibromo-1′,6′-dihydroxy-4′-oxocyclohex-2′-enyl) Acetonitrile (DT, obtained from *Pseudoceratina* sp.) induced a potent apoptotic response in K562 leukemic cells (2.5–10 μg/mL, 24 h) [117]. This effect coincided with elevated protein levels of p-AKT along with reduced p-PTEN (5 μg/mL, 6–18 h) [117]. A series of bioactive compounds (derived from *Chondrosia corticata*), namely halichondramide, jaspisamide A, halishigamide D, neohalichondramide, and (19Z)-halichondramide, were tested with respect to their effect on the cellular proliferation of an array of cancer cell lines (lung A549, colon HCT-116, breast MDA-MB-231, liver SK-HEP-1, and stomach SNU-601) [62]. (19Z)-Halichondramide (followed by halichondramide) was identified to be the most potent in A549 (with IC_50_ values of 0.024 μM) [62]. Along with changes in tubulin polymerization (via immunofluorescence staining), there was a reduction in p-AKT, p-mTOR, and p-p70S6K along with reduced levels of p-ERK and p-p38 proteins [62].

*Subclass of Keratosa:* Dysifragilone A (obtained from *Dysidea fragilis*) did not alter the viability of RAW264.7 macrophages (up to 100 μM, 24 h) [118]. With LPS treatment, the bioactive compound reduced the levels of iNOS and COX-2 while also reducing p-p38 protein levels (100 μM) [118]. 12-Deacetyl-12-epi-scalaradial (obtained from *Hippospongia* sp.) inhibited the cellular viability of HeLa cervical cancer cells (with IC_50_ values of 13.74 μM), associated with apoptotic characteristics as well as reduced p-ERK in the absence of changes in p-JNK, p-p38, and p-AKT [119].

***JAK/STAT:*** There were only two articles identified in reference to this pathway in sponges: one within the Heteroscleromorpha subclass and another within the Keratosa subclass.

*Subclass of Heteroscleromorpha:* Stellettin B (purified from *Jaspis stellifera*) inhibited K562, KU812, and U937 cellular viability (with IC_50_ values of 0.035 μM, 0.95 μM, and 4.55 μM, respectively) [120]. In K562 cells, this was associated with an apoptotic response as well as reduced p-STAT5 levels (the changes in p-STAT3 were unremarkable) amongst other signaling pathway alterations (e.g., p-PDK1, p-AKT, p-mTOR, and p-p70S6K) [120]. Moreover, siRNA targeting STAT5 led to a reduction in PI3K protein expression, implicating a link between the STAT5 and PI3K/AKT networks [120].

*Subclass of Keratosa:* 9,11-Dihydrogracilin A (DHG, purified from *Dendrilla membranosa*) was found to inhibit phytohemagglutinin (PHA) and CD3-monoclonal antibody (OKT3)-induced mitotic response (0.3–10 μM) in peripheral blood mononuclear cells (PBMCs) [121]. Along with reduced cytokine production (e.g., IL-6 and IL-10), this response was associated with reduced p-STAT5 levels (120 min) [121].

***Cell Surface Signaling Receptors:*** The majority of articles identified in reference to cell surface receptors in sponges were within the Heteroscleromorpha subclass with a smaller subset within the Verongimorpha and Keratosa subclasses.

*Subclass of Heteroscleromorpha:* Stellettin B (isolated from *Rhabdastrella* sp.) inhibited cellular viability in bladder cancer cell lines including RT-112 (0.02–0.16 μM), which was associated with apoptotic features [122]. Via a protein kinase array, p-FGFR3, p-EGFR, and p-ErbB2 were reduced at the protein level (0.125–0.5 μM, 24–48 h) along with reduced levels of p-AKT and p-STAT3 (downstream of FGFR signaling) [122]. Halenaquinone (purified from *Xestospongia carbonaria*) elicited the inhibition of cellular proliferation (EC_50_ values of 1–10 μM) across a series of cell lines, including RR1022 (rat cells transformed with Rous sarcoma virus), NY684 (rat cells transformed with a temperature-sensitive variant of v-src), NIH src (NIH 3T3 transformed with wild-type c-src), NIH erbB-HX (NIH 3T3 transformed with v-erbB), and CH^R^C5 (multidrug-resistant Chinese hamster ovarian cells) [123]. This bioactive compound also inhibited src tyrosine kinase activity (with an EC_50_ of 1.5 μM) and EGFR kinase activity (with IC_50_ values of 19 μM) [123]. Two bioactive compounds (purified from *Cribrochalina vasculum*), namely (3R)-icos-(4E)-en-1-yn-3-ol and (3R)-14-methyldocos-(4E)-en-1-yn-3-ol, were found to specifically target the IGF-1R signaling pathway [124]. Protein levels of p-IGF-1R (and p-IRS-1) were reduced in U-1810 non-small cell lung cancer cells [124]. In silico docking studies demonstrate that the binding site in the IGF-1R receptor is identical to the AG1024 tyrosine kinase inhibitor in the kinase domains using PDB (2ZM3) [124].

Aaptamine (purified from *Stylissa* sp.) caused diminished cellular viability in HCT116 colon cancer cells (a 50% reduction, 50 μM, 48 h) [125]. Furthermore, using a beta-arrestin assay for screening 169 G-protein coupled receptors (GPCRs) with aaptamine (20 μM) to uncover a pharmacological profile, 13 hits were obtained (CCR1, CXCR7, ADRA2A, ADRA2C, ADRB2, DRD2L, DRD2S, DRD4, CCR3, CXCR3, HTR1E, OPRK1, and SSTR1), which included alpha-adrenoreceptors, beta-adrenoreceptors, and dopamine receptors [125]. In a separate report, aaptamine (purified from another sponge, *Aaptos aaptos*) and its derivatives (9-demethyl aaptamine, demethyl (oxy)-aaptamine, fascaplysin, and 10-bromo-fascaplysin) were tested for their agonist activity towards the μ-OR (opioid receptor) and δ-OR [126]. These agents did elicit activity towards these GPCRs as determined by the cAMP accumulation, biotin protection assays, and beta-arrestin-2 recruitment in HEK293T cells [126].

*Subclass of Verongimorpha:* (+)-Aeroplysinin-1 (derived from *Verongia aerophoba*) reduced the EGF-stimulated proliferative capacity in MCF-7 breast cancer cells (0.5–0.25 μM) as well as EGFR endocytosis and EGFR kinase activity [127].

*Subclass of Keratosa:* A series of bioactive compounds (purified from *Spongionella* sp.), namely gracilin J, gracilin K, gracilin H, gracilin I, gracilin L, tetrahydroplysulphyrin-1, and 3′-norspongiolactone, elicited cell death in the K562 chronic myelogenous leukemia cell line (with IC_50_ values of 0.6–15 μM) [128]. This cellular response was associated with the potent inhibition of EGFR kinase activity (100 μM), with gracilin L being the most potent (a 75% inhibition) [128].

#### 4.2.4. Calcarea Sponge

*Subclass of Calcinea:* Naamidine A (NA, derived from *Leucetta chagosensis*) inhibited DNA synthesis in A-431 human epidermoid cancer cells (0.78 μM, 30 h) that were stimulated with EGF [129]. The induction of G1 phase arrest in the absence of features of cell death (no increase in the sub-G_0_ percentage) or PARP cleavage (apoptosis) was also noted [129]. Since EGF stimulation activates the MAPK signaling cascade, the effect of NA on MAPK activation was investigated [129]. In contrast to expected findings, the levels of phosphorylated ERK1/2 protein increased to higher levels in cells co-treated with EGF along with NA (0.78 to 3.13 μM) in contrast to EGF stimulation alone [129]. It is proposed that the sustained activation of ERK may support findings of its detrimental effect on cell health [129].

### 4.3. Gaps in Knowledge, Limitations, and Future Perspectives

**Figure 12:**
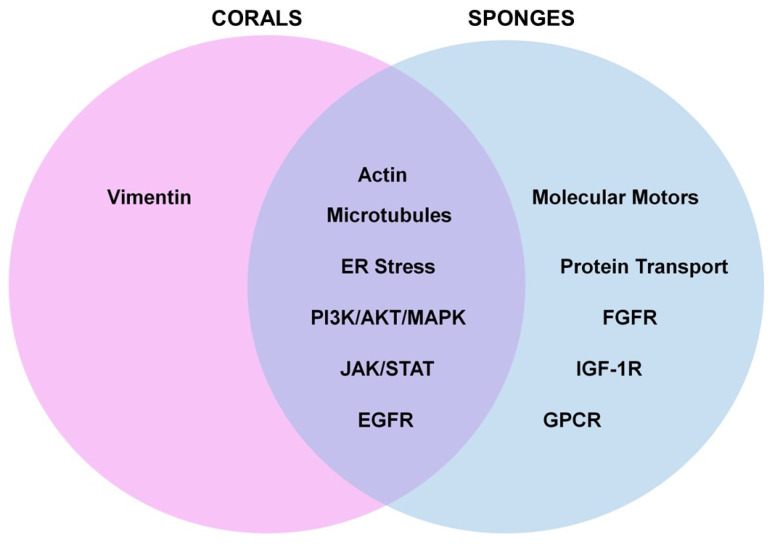
Venn diagram model presentation of similarities and differences between the pathways altered by bioactive compounds derived from corals and sponges. Commonalities between these two sessile invertebrates included deregulation of microtubules and actin filaments, induction of ER stress, activation of PI3K/AKT/MAPK, and JAK/STAT, and EGFR. Bioactive compounds from sponges also elicited bioactivities towards protein transport, intermediate filaments (such as vimentin), and other cell surface receptors including GPCR.

*Gaps in Knowledge:* The majority of the analyzed studies focused on marine metabolites (from both corals and sponges) that elicited activities as anti-cancer agents across a wide array of cancer types (solid tumors and those derived from the hematopoietic system). A smaller subset of studies focused on bioactivities relating to neuroprotection, the inhibition of coronaviral protease, anti-inflammation, liver disease, fungal infections, angiogenesis, and UVB-induced skin aging. As summarized in Figure 12, commonalities between these two sessile invertebrates included the deregulation of microtubules and actin filaments, induction of ER stress, activation of signaling networks such as PI3K/AKT/MAPK and JAK/STAT, and receptor activation (e.g., EGFR). However, bioactive compounds derived from sponges additionally elicited activities with respect to targeting protein transport/post-translational modifications and activities towards other cell surface receptors, including GPCR, FGFR-1, and IGF-1R.

Similar to current knowledge regarding bioactive compounds derived from the *Ericaceae*, *Rosaceae*, and *Cucurbitaceae* plant families [130], the majority of the studies described herein for coral- and sponge-derived products were also descriptive in nature (e.g., the protein alterations were noted via immunofluorescence, via Western blot analyses, or via profiling studies) and associated with a functional response (e.g., cell death, reduced cellular proliferation, reduced migration, etc.). Likewise, only a subset of these studies investigated the contribution of the protein level alterations to the observed functional response, using specific inhibitors or siRNA-based approaches. However, a few reports investigated the molecular mechanism of action of a few bioactive compounds including interactions with the coronaviral main protease (M^pro^, via molecular docking studies), EGFR (via molecular docking studies), the binding region on β-tubulin (via in vitro and molecular docking studies), the binding region on molecular motors (via in vitro assays), or the modulation of specific enzyme isoforms such as PI3K (via in vitro enzyme assays). With respect to protein movement and maturation, specialized transport assays were performed to demonstrate that a sponge-derived bioactive compound could modulate Golgi integrity. While current knowledge with respect to the mechanism of action of these marine metabolites is broad, their precise mechanism of action is worthy of further investigation.

*Limitations:* Deep-sea sponges and corals are under-researched marine organisms with immense potential for contributing to the drug discovery process. Further research is needed to investigate the bioactivities of metabolites from deep-sea sponges and corals located off the western coast of Florida; none were identified from our analyses described above. The use of specific search terms is likely to have led to the omission of a number of relevant articles. In addition to limitations with the PubMed searches performed, several NOAA entries had incomplete annotations; furthermore, it is likely that corals and sponges exist within the region of interest that were not represented within the map extent presented in Figure 1 and Figure 3.

*Future Perspectives:* To combat the rising issue of overharvesting and inadequate marine bioactive drug supply, technological advancements have been developed to cultivate marine sponges and corals to support the drug discovery pipeline. These advances include ex situ and in situ culturing, the establishment of long-term cell cultures and cell lines, and symbiont cultivation, for which evidence is available to support their sustainability for both corals and sponges. This discussion is presented below in Section 5.

## 5. Supporting the Drug Discovery Pipeline

### 5.1. Overview—“Supply Issue” and Methodological Solutions

Since the 1960s, more than 40,000 marine natural compounds have been uncovered according to the MarineLit database (https://marinlit.rsc.org (accessed on 13 November 2023 through the 18 November 2023)) [131,132]. As stated earlier, the majority of bioactive compounds from marine sources are derived from sponges [132,133,134,135]. These compounds include terpenoids, alkaloids, polysaccharides, and polyphenols, amongst others [136]. While the purpose of marine-derived compounds is primarily to provide protection against predators [137] and fouling agents [138], they have potential as pharmaceuticals for treating human diseases, such as cancer [136]. Regrettably, the productive isolation of natural products from sponges and corals is negatively affected by environmental stressors [139]. Given the consequences of such stressors to the reduced production of such compounds [139], the development of sustainable culturing methodologies are essential to support not only the protection and survivability of these sessile marine invertebrates within their natural environment but also the drug discovery pipeline. Thus, the overall goal for this field of research field is to increase organismal biomass (e.g., of sponges or corals) within controlled environments [140,141].

Although a chemical synthesis procedure has been successfully applied for generating halichondrin (derived from *Halichondria okadai*, whose synthetic derivative successfully made its way to Phase II [142] and Phase III clinical trials [143]), such chemical methods are costly and labor-intensive due to the complexity of the metabolite structure [144]. Unfortunately, genetic engineering methodologies have yet to be fully developed and optimized for use in marine invertebrates [145,146,147]. This is unlike methods that are well established and feasibly implemented in mammalian model systems. It has been suggested that partial or entire pathways could be expressed in hosts (e.g., *E. coli*) to produce these metabolites [145]. However, knowledge of a fully sequenced genome to identify responsible enzymes for bioactive compound generation is required but is expected to have a high potential to propel the field forward [145,148]. Other goals are to create immortal sponge and coral cell lines, develop efficient transfection methodologies, and identify a suitable sponge promoter [147]. Sponge promoters from *S. domuncula* have displayed strong activity when expressing fluorescent proteins in 3T3 mammalian cells [147]. Additionally, transfection was successfully performed into primmorphs using a microprojectile method with a plasmid containing a CMV promoter [147,149].

The symbiotic relationship between symbionts and marine invertebrates adds another layer of complexity to tackling the “supply issue” [148]. Genetic analyses of sponge–microbial interactions, referred to as metagenomics, may help unravel such organismal association complexities [148,150]. While this strategy is likely to have a high value in future efforts, the application of this method to sponge- or coral-derived natural products has not yet been reported, with the exception of the polyketide synthesis pathway [151]. Furthermore, uncovering the cell origin of bioactive compounds in marine invertebrates remains important for defining the cultivation system for use in large-scale production (e.g., aquaculture, cell culture, or the cultivation of symbionts) [152].

**Figure 13:**
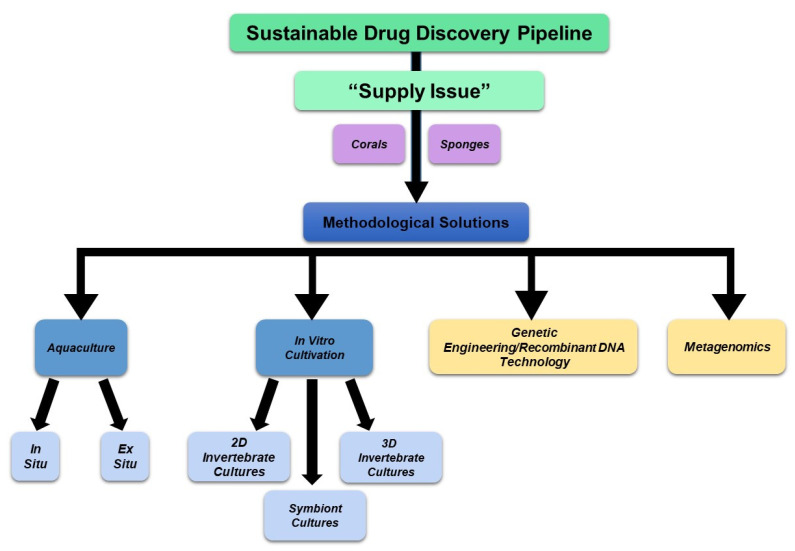
Schematic of technological advancements to overcome the “supply issue” and sustain the drug discovery pipeline. Methods involving aquaculture and in vitro cultivation have been developed and applied to specific species of sponges and corals. Future advancements are needed in the fields of metagenomics and genetic engineering/recombinant DNA technology, which are presently limited.

Successfully implemented techniques in this field that are relevant to the optimal production of specific bioactive compounds include mariculturing (*in situ* aquaculture), in vitro three-dimensional (3D) sponge primmorph cultures under controlled conditions, the cultivation of sponge and corals within controlled environments, the establishment of new cell lines, explant sponge farming, and the propagation of larvae or gemmules [146]. We have discussed several of these approaches in further depth below and a schematic of these approaches is summarized in Figure 13. In addition, from the literature reviewed, the scientific name, depth of collection, season and year of collection, and relevant details pertaining to the metabolite tested (extract, purified, or synthesized form) are summarized in Supplementary File S16 and Supplementary File S17, for sponges and corals, respectively.

### 5.2. Aquaculture

Cultivation methods in this category can be classified into two major approaches for marine sponges: (a) in situ (also referred to as mariculture) and (b) ex situ [1,6,146]. These methods of aquaculture may offer approaches towards the long-term stable production of bioactive compounds [146].

*In situ Mariculture:* In situ nurseries (i.e., sea-based cultures located in the natural habitat of the marine invertebrate) are sustainable methods for coral preservation and culturing to support future research [1,153], with comparatively lower maintenance and equipment costs [153]. In this cultivation approach, explants of sponges are placed on man-made substrates, such as glue, wood, cages, or rope [146,154], and then returned to their natural environment. However, careful planning is needed to minimize the organisms’ sensitivity to environmental conditions, including sedimentation, temperature, salinity, and pH [153,155,156,157], to optimize their growth. Altogether, whether or not cultivation is successful is dependent on the optimal environmental conditions since it is well established that environmental stressors (e.g., predators, fouling, diseases, inclement weather, and climate change leading to bleaching events) [146,158,159,160,161] can negatively affect growth and survivability. The inability to appropriately regulate such environmental parameters in the mariculture system has been shown to negatively affect the coral growth rate [153]. With regard to the sponge–symbiont populations, changes in temperature [159,162], heavy metal exposure [159,163], and diseases [159,164] (as well as movement from the natural habitat to containments [159,165,166]) can be detrimental to the health of marine invertebrates.

Altogether, the in situ farming of explants appears to be a viable approach to maintaining the delicate ecological balance while potentially supporting the production of metabolites in large quantities in a cost-effective manner.

*Ex situ Aquaculture:* The major advantage of this cultivation method over the in situ nursery is that it allows researchers to modify critical environmental parameters, such as temperature, light, and water flow, to optimize growth rates [167]. To overcome environmental challenges, marine invertebrates (i.e., sponges) have been placed into containments (i.e., aquariums) in which the environmental conditions are regulated and can be optimized for different species [146,154]. Therefore, ex situ refers to the practice of cultivating organisms outside their natural habitat, in controlled environments [153]. This is one of the most effective types of aquaculture for supporting the drug discovery pipeline due to organismal maintenance under a controlled and stable environment with optimal conditions for their growth and metabolite production [1]. Ex situ aquaculture involves the collection of, for example, corals from the natural environment and their placement into tanks containing abiotic and biotic factors to support their optimal growth and the production of bioactive compounds [3]. Specific ex situ coral cultivation methodologies have been reported that utilize flow-through systems (FTS) incorporating seawater derived from the natural environment [167]. Moreover, ex situ approaches can be implemented with autotrophic/heterotrophic feeding methods [3,167], which improve growth rates. For example, the effect of light on heterotrophic feeding using a recirculating aquaculture system (RAS) on *Pocillopora acuta* resulted in a viable culture for a prolonged time period (140 days) with a four-fold size expansion under intense light and with shrimp feeding [167]. Such results highlight the potential for ex situ cultivation to support coral biomass expansion for drug discovery.

Since dissolved oxygen, balanced light exposure, and waste removal are critical elements that modulate the survivability of these marine organism [158], these must be carefully regulated. In addition, water flow can affect the efficiency of sponge feeding as currents are necessary for food transport [6]. Other important considerations include the microbial diversity of the sponge which may affect its cultivation success [168]. Specifically, the transfer of sponges to aquarium tanks may alter bioactive compound production due to changes in the marine invertebrates’ microbiome [146,169,170,171]. An element of stress has also been proposed to contribute to the changes in microbial population and thus must be considered [166].

*Ex situ* up-scaling is thought to be onerous. As coral fragments get larger and the nursery culturing time increases, labor, maintenance, and space become a problem [153]. The significant energy requirement is an important consideration (e.g., pumping water and other essential utilities) [172]. While ex situ provides an array of advantages to support the development of a successful long-term coral culture, growing such sessile organisms outside of their natural habitats is associated with higher costs.

### 5.3. In Vitro Cultivation

The lack of knowledge regarding cell nutrition requirements for in vitro cultivation has challenged long-term sponge and coral viability [173]. In a recent study involving the in vitro cultivation of *Crambe crambe* explants, octopus extract (used at 5–20%) supported their growth and metabolic activities when added together with amino acids, lipids, vitamins, oligoelements, hormones, growth factors, and cell adhesion molecules [174]. Furthermore, the supplementation of Leibowitz L15 media with chick embryo extract, calcium ionophore, and phorbol esters (TPA) positively altered *Negombata magnifica* sponge culture viability [175]. Iron and silicate along with factors such as myotrophin-like polypeptides within the nutrient medium may also help the cultivation succeed [147]. A genetic algorithm strategy was recently employed to test a wide array of components for the optimization of amino acids in culture media for supporting sponge cell cultures [176]. In a recent 2023 study, this genetic algorithm approach was used to develop a continuous cell line from *Geodia barretti* [177]. In this study, a new culture medium, named OpM1, was developed, containing (a) trace elements (sodium metasilicate and zinc sulfate), (b) a vitamin mixture (sodium ascorbate and sodium pyruvate), (c) a lipid mixture, (d) insulin-transferrin-selenium-ethanolamine, (e) fetal bovine serum, (f) pifithrin-α, (g) platelet-derived growth factor, and (h) a growth factor mixture (epidermal growth factor, insulin-like growth factor-1, and phytohemagglutinin (PHA)), in Roswell Park Memorial Institute (RPMI) 1640 base [177]. One of the vital components that increased media performance was PHA, which increased the proliferation rate and led to population doubling [177]. Cryopreserved cells could also be released with high viability to restart cultures [177]. Such a genetic algorithm strategy could be utilized in the future to identify the requirements for other components, such as vitamins, elements, and growth factors.

Other obstacles have included tissue dissociation methods and contamination from rapidly growing microorganisms. With respect to tissue dissociation, two approaches exist, including a destructive approach (affecting tissue organization) and a non-destructive approach (preserving organization) [178]. Destructive approaches involve the reduction of calcium to break down the cells’ extracellular matrix adhesion which diminishes tissue viability and increases microbial contamination [178]. For stony corals, calcium- and magnesium-free seawater, as a non-destructive approach, aids cellular release from the skeleton by interfering with cell–cell interactions [5]. Other non-destructive approaches include mechanical methods that peel coral tissues or that induce polyp bailout; these increase coral viability and decrease contamination [178].

Furthermore, for coral cultures, the best cultivation time is suggested to be during the winter season to support optimal aggregation formation at ~12 °C under controlled light/dark conditions, such as that reported for *Eunicella singularis* [179]. Tissue fragments could also be utilized for in vitro culture systems to minimize the stressful consequences of cellular dissociation [146]; however, the use of this strategy has been limited. With regards to in vitro sponge cultures, stem cells offer extensive proliferative potential and the ability to differentiate to other cell types [146,180]. Other considerations include the symbionts that associate with these invertebrates, which may actively participate in the host defense pathways with respect to bioactive metabolite production [181].

*Invertebrate 2D Cell Cultures:* An infinite cell line would greatly improve research efforts in the sustainable generation of bioactive metabolites. While there has been much effort to generate such a cell line, establishing one has not been successful due to the decline in cell viability and proliferative capacity of isolated cells [180]. Until 2021, there was no report on any coral cell or tissue surviving for more than 13 days in a culture [160]. Altogether, an immortal cell line has not yet been obtained for sponges, but when developed, it could be applied to the use of photobioreactors for the large-scale production of a compound of interest [148]. Unambiguous cell type identification along with culture media optimization also requires further efforts [182]. 

Obstacles in the successful development of an immortal sponge cell line are attributed to problems with cells in the non-growth phase or cells that have differentiated [135]. Unfortunately, most sponge cells derived from adult organisms are already in the differentiated state, and thus this cellular characteristic will limit their proliferative capacity. The use of larvae or gemmules as well as minimally invasive polyp bailout may be key in achieving immortal coral cell lines [160]. Polyp bailout is a process in which coral polyps detach from a larger coral colony to live as independent individuals [160]. This novel method for coral cell isolation for propagation is suggested to improve culture viability [160]. Studies on small (0.5–2.5 mm) tissue explants from *Fungia granulosa* demonstrated that properly dissected coral tissue may be propagated to enable the induction of polyps (forming mouths, septae, tentacles, and calcium carbonate skeletons) [183]. Fragmented coral tissues develop into larvae-like “tissue balls” which can be maintained for over three months in culture [184]. These regenerative properties may be advantageous for improving coral cell culture techniques and preserving species [183,184].

The current knowledge that sponges have stem cells may propel immortal cell line development. Stem-cell-like cell lines are preferred because they may produce persistent cell lineages [178]. Tropical corals have been a point of focus because of their longer-than-usual lifespan, and stem-cell-like cells have been found and characterized in Hydrozoa cnidarians as interstitial cells, meaning they are located in between ectodermal cells [178].

Initial steps in the derivation of sponge cultures involve dissociation methodologies that can include mechanical, chemical, or spontaneous techniques [146]. Regardless of the specific dissociation method used, a mixed pool of different cell types (e.g., stem-like cells such as archaeocytes and choanocytes, which can be further purified) is initially obtained; this initial cell material contains variant cell numbers with different levels of viability [146,182]. The identification of optimal media conditions has been one of the major obstacles to successfully establish sponge and coral cell lines. As indicated earlier, it is well established that mammalian cell media are not ideal for marine invertebrate cell lines [185,186]. Additionally, contamination is an obstacle to the establishment of sponge cell cultures. While these may arise from any of the sponge holobionts, limiting this contamination is challenging. Although antibiotics and antimycotics can be utilized, these treatments negatively affect the survivability of the sponge cell cultures [175]. One positive media adaptation is the use of filtered seawater [146,187], which can antagonize the growth of these holobionts. With corals, there is a major lack of understanding in the interaction between corals and microbials, which may be necessary in developing a medium suitable for long-term coral cultures and cell lines [178].

*Primmorph 3D Cell Cultures:* This technology developed for sponges is relatively novel. Primmorphs are cell aggregates that form within 5 days (up to 5 mm in diameter) characterized by a proliferating cell population comprised of archeocytes, spherulous, or amoebocytes [147]. The successful formation of these sphere-like balls may depend on the initial seeding density [146,188]. The long-term proliferative capacity of sponge cells in vitro can be assessed using the primmorph system [189,190,191]. Primmorph cultivation is successful and evidence supports the idea that dissociated single cells lose their ability to divide [189]. Sponge primmorphs’ success is comparable to optimized in vitro cultures for octocorals [179]. Primmorphs retain their ability to proliferate and recover telomerase activity, as compared to monodisperse cells, which lose these critical characteristics following dissociation [146], possibly due to loss of cell–cell and cell–matrix contacts [146]. While some species appear to be more conducive to primmorph formation, there are varied reports in the literature and this may be due to the season of collection [146]. 

A new approach to in vitro sponge primmorph cultures based on an archeocyte-dominant cell population (ADCP) utilized totipotent archaeocytes [192]. Archaeocytes have stem-cell-like properties, and thus this cell type is proposed for use in in vitro cell culture development [182]. Totipotent archaeocytes are thought to be better equipped in maintaining their proliferative potential in vitro compared to traditional mixed-cell populations (MCPs) isolated from sponges [192], as reported for the sponge *Hymemicidon perleve*. This study successfully demonstrated the proliferative capacity of ADCPs in primmorphs, enabling the generation of increased biomass [192]. 

Since primmorphs are 3D sponge cell cultures and contain symbionts [193], another method for sustainable bioactive compound production is to optimize the growth of the sponge-associated bacteria, while also optimizing the primmorph culture model [193]. Interestingly, different substrata were shown to strongly influence sponge primmorph growth [194]. The three-dimensional shape of *Petrosia ficiformis* primmorphs developed more rapidly with quartz than marble [194]. Utilizing quartz to increase growth rates should be explored in future research optimizing technological advancements in these culturing models [194]. Additional contributors to successful primmorph generation are the presence of silica and iron [146,189]. For example, in a study of wild-type *Suberites domuncula* demosponge, one impediment in obtaining primmorphs greater than >10 mm was the restricted oxygen supply [189]. To overcome this limitation and enable canal formation to support both oxygen and nutrient uptake, the researchers utilized a medium containing silicate and iron [189]. This primmorph technique was applied to another sponge species, namely *Dysidea avara*, which produced high concentrations (equivalent to those produced from those in the wild) of avarol, a bioactive compound with antitumor/antibacterial/antiviral activities [189].

A scale-up of this primmorph technique would be necessary to enable the greater abundance of bioactive compounds; in this respect, genetic engineering methods could be integrated with this specific cell culturing method to enable supply-chain productivity levels in future approaches.

*Cultures of Symbionts:* Symbionts (e.g., bacteria, archaea, and fungi) [146] comprise roughly 60% of the total biomass of sponges [169,195]. The roles of symbionts with sponge hosts include photosynthetic carbon [169,196] and nitrogen fixation [169] as well as defense against predators, contaminants, and fouling agents [137,146,169,197]. In corals, dinoflagellates support respiration for coral growth [185]. Shallow water corals have symbiotic relationships with *Symbiodiniaceae* algae, which provide additional energy to support coral functions [160]. Coral bleaching is due to the loss of these symbiotes [160,161].

The symbiotic relationship between corals or sponges with their own symbionts is of critical importance with respect to the generation of bioactive compounds. Therefore, further knowledge of the metabolic enzymes/pathways as well as the regulators responsible for bioactive compound production is needed [148]. However, isolating sponge-associated microorganisms is notoriously difficult, as many cells will not grow outside of their normal environment, as changes in the environment can alter the bacterial symbionts associated with the host, including changes in temperature [159,162], heavy metal exposure [159], diseases [159,164], and other aspects, for example, when moving wild-type sponges to an in situ aquarium system [159,166]. Methods to identify bacterial symbionts include deep pyrosequencing and culture-based techniques [198]. For example, bacterial culturing from two sponge species, *Xestospongia muta* and *Xestospongia testudinaria*, identified 50 genera, including *Actinobacteria*, *Firmicutes*, *Gammaproteobacteria*, *Alphaproteobacteria*, and *Bacteriodetes* [198]. Nonetheless, the relationships between these sessile invertebrates and their symbionts are critical in advancing technologies to cultivate the host cells.

### 5.4. Future Considerations

*Coral–Sponge Interactions:* While the data and analyses presented herein are focused on coral and sponge marine invertebrates independently from each another, careful consideration to their overlapping niches and interactions within their native ecosystems is required. It is well known that coral reefs provide a refuge for an array of organisms including sponges, which may alter the dynamics of the reef [199]. Lamentably, the consequences of global climate change, pollution, diseases, and other impacts are seen worldwide on the coral reef ecosystem with respect to its ecological interactions with other marine organisms including sponges [199,200]. Both sessile invertebrates feed on partially overlapping food types; therefore, this may lead to competition on existing resources, if these become limited [201]. Some species of sponge are considered invasive and cause coral death due to chemicals released from the sponge causing the degeneration of coral tissues [199]. Altogether, consideration of coral–sponge interactions (with other reef organisms) may be needed to support ex situ cultivation in future efforts.

*Aquaculture Opportunities off the Western Coast of Florida:* In 2019, a partnership began between NCCOS (NOAA’s National Centers for Coastal and Ocean Science) and FDACS (Florida Department of Agriculture and Consumer Services) [202]. The goal of this partnership was to examine coastal regions off a segment of the western coast of Florida for their development potential as aquaculture zones (namely, POAZs). Upon the completion of the study, 34 sites were identified with sizes ranging between 204 and 7407 acres (with a total coverage area of 54,904 acres) [202]. This area spanned an area between Sarasota and Pensacola Florida [202]. The sites that were identified utilized analysis strategies to minimize potential impacts to the existing marine ecosystems [202]. Collectively, these analyses will enable FDACS to work with state and federal agencies to identify the best sites to support the future development of aquaculture.

## Figures and Tables

**Table 1:** Corals and sponges identified at specific sea depths from NOAA. The scientific name, vernacular name, number of NOAA entries, and sea depth category (specified in the blue highlights) (as per the Venn diagram in Figure 2) identified within the map extent presented in Figure 1 are presented in tabular format.

Scientific Name	Vernacular Name	Number of Records in NOAA	Venn Diagram Category
			**Depth < 50 m**
*Biemna*	Demosponge	1	A
*Cinachyra*	Demosponge	1	A
*Haliclona (Reniera)*	Demosponge	1	A
*Leptogorgia hebes*	Gorgonian coral	1	A
*Phacelocyathus flos*	Stony coral (cup coral)	1	A
*Phyllangia americana*	Stony coral (cup coral)	9	A
*Phyllangia americana americana*	Stony coral (cup coral)	1	A
*Phyllangia americana mouchezii*	Stony coral (cup coral)	4	A
*Stelletta kallitetilla*	Demosponge	1	A
			**Depth ≥ 50 to <200 m**
*Aaptos pernucleata*	Demosponge	2	B
*Aiolochroia crassa*	Demosponge	2	B
*Antho (Acarnia)*	Demosponge	1	B
*Antipathes*	Black coral	16	B
Antipathidae	Black coral	3	B
*Aplysina*	Demosponge	1	B
*Aplysina* cf. *insularis*	Demosponge	1	B
*Aplysina fistularis*	Demosponge	1	B
*Asteropus*	Demosponge	1	B
*Axinella*	Demosponge	1	B
*Axinella corrugata*	Demosponge	3	B
*Axinella shoemakeri*	Demosponge	2	B
*Balanophyllia (Balanophyllia) floridana*	Stony coral (cup coral)	1	B
*Bebryce*	Gorgonian coral	3	B
*Bubaris*	Demosponge	2	B
*Callyspongia (Cladochalina)*	Demosponge	1	B
*Caryophyllia (Caryophyllia) horologium*	Stony coral (cup coral)	4	B
cf. *Leucetta*	Calcareous sponge	3	B
cf. *Leucosolenia*	Calcareous sponge	10	B
*Chelonaplysilla*	Demosponge	2	B
*Chondrilla*	Demosponge	1	B
*Chondrilla nucula*	Demosponge	1	B
*Chondrosia*	Demosponge	1	B
*Chondrosia reniformis*	Demosponge	2	B
*Cinachyrella alloclada*	Demosponge	7	B
*Cinachyrella arenosa*	Demosponge	1	B
*Cirrhipathes*	Black coral	2	B
*Cladocora arbuscula*	Stony coral (branching)	1	B
*Cladocora debilis*	Stony coral (branching)	1	B
*Clathria*	Demosponge	1	B
*Clathria (Microciona)*	Demosponge	5	B
*Cliona*	Demosponge	5	B
*Cliona varians*	Demosponge	14	B
*Dasmosmilia lymani*	Stony coral (cup coral)	1	B
*Desmacella*	Demosponge	1	B
*Diodogorgia nodulifera*	Gorgonian coral	2	B
*Dragmacidon reticulatum*	Demosponge	1	B
*Dysidea*	Demosponge	24	B
*Elatopathes*	Black coral	7	B
*Ellisella*	Gorgonian coral	6	B
*Ellisella atlantica*	Gorgonian coral	1	B
*Ellisella elongata*	Gorgonian coral	1	B
Ellisellidae	Gorgonian coral	1	B
*Epipolasis*	Demosponge	5	B
*Erylus trisphaerus*	Demosponge	2	B
*Eurypon*	Demosponge	1	B
*Flabellum (Flabellum) floridanum*	Stony coral (cup coral)	4	B
*Funiculina quadrangularis*	Sea pen	1	B
*Geodia*	Demosponge	2	B
*Geodia gibberosa*	Demosponge	2	B
*Halichondria*	Demosponge	6	B
*Halichondria (Halichondria) melanadocia*	Demosponge	3	B
*Haliclona*	Demosponge	2	B
*Haliclona (Gellius)*	Demosponge	1	B
*Higginsia*	Demosponge	1	B
*Higginsia strigilata*	Demosponge	1	B
*Hymedesmia*	Demosponge	1	B
*Hymeniacidon caerulea*	Demosponge	1	B
*Hypnogorgia pendula*	Gorgonian coral	12	B
*Igernella notabilis*	Demosponge	1	B
*Ircinia*	Demosponge	4	B
*Ircinia campana*	Demosponge	1	B
*Ircinia felix*	Demosponge	1	B
*Ircinia strobilina*	Demosponge	1	B
*Jaspis*	Demosponge	1	B
*Leptogorgia cardinalis*	Gorgonian coral	2	B
*Leptogorgia stheno*	Gorgonian coral	2	B
*Leucetta*	Calcareous sponge	1	B
*Leuconia*	Calcareous sponge	1	B
*Lissodendoryx (Anomodoryx) sigmata*	Demosponge	1	B
*Madracis*	Stony coral (branching)	4	B
*Madrepora oculata*	Stony coral (branching)	3	B
*Mycale (Mycale) laevis*	Demosponge	4	B
*Neofibularia nolitangere*	Demosponge	1	B
*Nicella*	Gorgonian coral	7	B
*Nicella guadalupensis*	Gorgonian coral	1	B
*Nicella toeplitzae*	Gorgonian coral	1	B
*Nidalia*	Soft coral	1	B
*Oculina diffusa*	Stony coral (branching)	3	B
*Oculina tenella*	Stony coral (branching)	3	B
*Oxysmilia rotundifolia*	Stony coral (cup coral)	1	B
*Placospongia melobesioides*	Demosponge	3	B
*Poecillastra*	Demosponge	3	B
*Riisea paniculata*	Gorgonian coral	1	B
*Scopalina ruetzleri*	Demosponge	1	B
*Siphonodictyon siphonum*	Demosponge	2	B
*Spheciospongia*	Demosponge	1	B
*Spheciospongia vesparium*	Demosponge	3	B
*Spirastrella*	Demosponge	1	B
*Spirastrella coccinea*	Demosponge	5	B
*Stelletta*	Demosponge	4	B
*Stelletta* cf. *grubii*	Demosponge	1	B
*Stichopathes luetkeni*	Black coral	1	B
*Stylaster*	Lace coral	1	B
*Swiftia exserta*	Gorgonian coral	2	B
*Telesto sanguinea*	Stoloniferan coral	8	B
*Terpios*	Demosponge	2	B
*Tethya*	Demosponge	2	B
*Tethya actinia*	Demosponge	1	B
*Tethya seychellensis*	Demosponge	4	B
*Thesea*	Gorgonian coral	2	B
*Timea*	Demosponge	3	B
*Timea mixta*	Demosponge	1	B
*Trochocyathus (Trochocyathus) rawsonii*	Stony coral (cup coral)	1	B
			**Depth ≥ 200 to <1000 m**
*Acanella aurelia*	Gorgonian coral	1	C
Acanthogorgiidae	Gorgonian coral	1	C
*Aphrocallistes*	Glass sponge	2	C
*Balticina*	Sea pen	1	C
*Bathypathes pseudoalternata*	Black coral	2	C
*Bathypsammia*	Stony coral (cup coral)	1	C
*Caryophyllia (Caryophyllia) ambrosia ambrosia*	Stony coral (cup coral)	1	C
*Deltocyathoides stimpsonii*	Stony coral (cup coral)	1	C
*Deltocyathus*	Stony coral (cup coral)	1	C
*Desmophyllum pertusum*	Stony coral (branching)	7	C
*Flabellum (Ulocyathus) moseleyi*	Stony coral (cup coral)	13	C
*Fungiacyathus (Bathyactis) crispus*	Stony coral (cup coral)	1	C
*Fungiacyathus (Bathyactis) marenzelleri*	Stony coral (cup coral)	1	C
*Leiopathes glaberrima*	Black coral	3	C
*Paracyathus pulchellus*	Stony coral (cup coral)	1	C
*Plumarella*	Gorgonian coral	4	C
Stylasteridae	Lace coral	14	C
*Tethocyathus recurvatus*	Stony coral (cup coral)	1	C
			**Depth ≥ 1000 to 3000 m**
Anthomastinae	Soft coral	4	D
*Caryophyllia (Caryophyllia) ambrosia*	Stony coral (cup coral)	1	D
Chrysogorgiidae	Gorgonian coral	1	D
Cladorhizidae	Demosponge	2	D
Coralliidae	Gorgonian coral	15	D
*Enallopsammia*	Stony coral (branching)	5	D
*Euplectella*	Glass sponge	9	D
Euplectellidae	Glass sponge	4	D
Farreidae	Sponge (unspecified)	1	D
*Heteropathes*	Black coral	3	D
*Hyalonema*	Glass sponge	1	D
*Iridogorgia*	Gorgonian coral	47	D
*Lepidisis*	Gorgonian coral	1	D
*Metallogorgia*	Gorgonian coral	6	D
*Narella*	Gorgonian coral	2	D
*Narella spectabilis*	Gorgonian coral	1	D
*Paragorgia*	Gorgonian coral	4	D
Paragorgiidae	Gorgonian coral	1	D
*Parantipathes*	Black coral	1	D
*Polymastia*	Demosponge	3	D
Primnoidae	Gorgonian coral	27	D
Scleractinia	Stony coral (cup coral)	5	D
*Stephanocyathus (Stephanocyathus) diadema*	Stony coral (cup coral)	3	D
*Tetilla*	Demosponge	1	D
*Umbellula*	Sea pen	4	D
*Victorgorgia*	Gorgonian coral	1	D
			**Depth < 50 m and Depth ≥ 50 to <200 m**
*Clathria (Thalysias) juniperina*	Demosponge	3	E
*Leptogorgia euryale*	Gorgonian coral	7	E
*Leptogorgia medusa*	Gorgonian coral	4	E
*Lissodendoryx*	Demosponge	4	E
*Madrepora*	Stony coral (branching)	12	E
Oculinidae	Stony coral (branching)	2	E
			**Depth ≥ 50 to <200 m and Depth ≥ 200 to <1000 m**
*Deltocyathus calcar*	Stony coral (cup coral)	6	F
Dendrophylliidae	Stony coral (unspecified)	4	F
*Mycale*	Demosponge	4	F
*Pennatulacea*	Sea pen	3	F
*Schizocyathus fissilis*	Stony coral (cup coral)	8	F
			**Depth ≥ 200 to <1000 m and Depth ≥ 1000 to 3000 m**
*Acanella*	Gorgonian coral	14	G
*Acanella arbuscula*	Gorgonian coral	12	G
*Anthomastus*	Soft coral	10	G
*Bathypathes*	Black coral	77	G
*Caryophyllia (Caryophyllia) ambrosia caribbeana*	Stony coral (cup coral)	9	G
*Chondrocladia*	Demosponge	3	G
*Chrysogorgia*	Gorgonian coral	10	G
*Chrysogorgia elegans*	Gorgonian coral	21	G
*Deltocyathus italicus*	Stony coral (cup coral)	13	G
Demospongiae	Demosponge	41	G
Hexactinellida	Glass sponge	63	G
Keratoisididae	Gorgonian coral	125	G
*Muriceides*	Gorgonian coral	6	G
			**Depth ≥ 50 to <200 m, Depth ≥ 200 to <1000 m, and Depth ≥ 1000 to 3000 m**
Antipatharia	Black coral	6	J
*Paramuricea*	Gorgonian coral	39	J
*Stichopathes*	Black coral	239	J
			**Depth < 50 m, Depth ≥ 50 to <200 m, Depth ≥ 200 to <1000 m, and Depth ≥ 1000 to 3000 m**
Porifera	Sponge (unspecified)	100	M
			**Depth ≥ 50 to <200 m and Depth ≥ 1000 to 3000 m**
Alcyonacea	Gorgonian coral	12	O
Caryophylliidae	Stony coral (unspecified)	2	O
Plexauridae	Gorgonian coral	12	O
*Swiftia*	Gorgonian coral	13	O
*Tanacetipathes*	Black coral	10	O

## Data Availability

All the data are presented within the manuscript files associated with the manuscript text.

## Supplementary Materials

The supporting information can be downloaded at: https://www.mdpi.com/article/10.3390/md21120615/s1.
