# Supplementary material for: Deep-Sea Sponges and Corals off the Western Coast of Florida—Intracellular Mechanisms of Action of Bioactive Compounds and Technological Advances Supporting the Drug Discovery Pipeline
Source: Mar Drugs. 2023 Nov 28;21(12):615. doi: 10.3390/md21120615 (PMC10744787; doi:10.3390/md21120615)
Supplement: Supplementary file 1 [file marinedrugs-21-00615-s001.zip › Supplementary_File_S14.pdf]

Marine Source	Compound	Structure	Compound ID
Coral	Eleutherobin	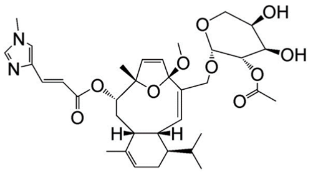	L10065
Coral	5-Epi-sinuleptolide	Not found	
Coral	Sinularin	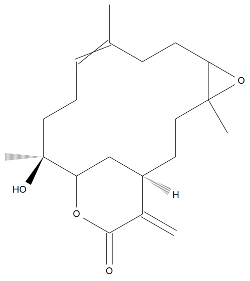	L2224
Coral	11-Dehydrosinulariolide	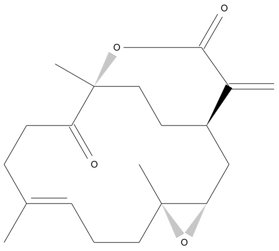	L2079
Coral	Sinulariolide	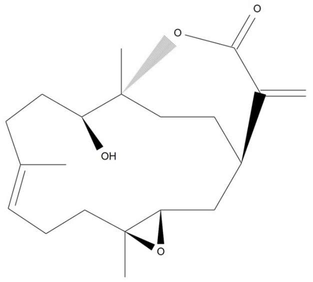	L1962

Coral	7-Acetylsinumaximol B	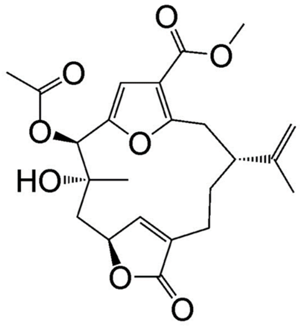	L28210
Coral	11-Epi-sinulariolide acetate	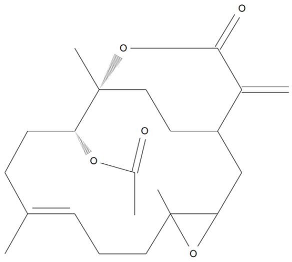	L2115
Coral	4-Carbomethoxy-10-epigyrosanoldie	Not found	
Coral	Bromovulone III	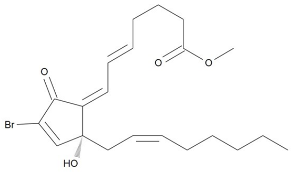	L15061
Coral	Crassolide	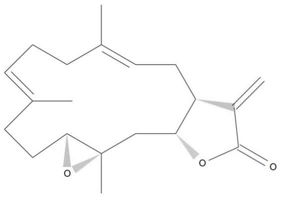	L12696

Coral	Flaccidoxide-13-acetate	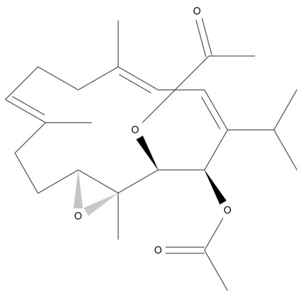	L12573
Coral	13-Acetoxysarcocrassolide	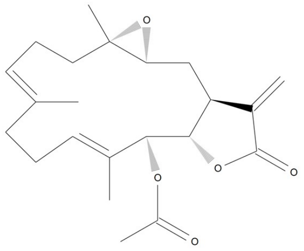	L12697
Coral	Methyl sartortuoate	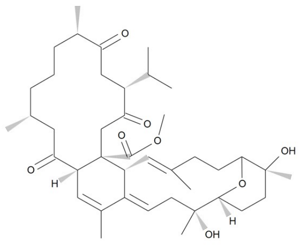	L17268
Coral	Sarelengan B	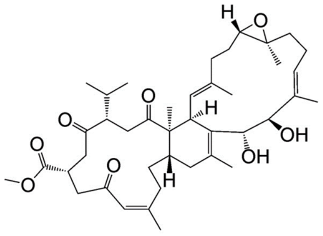	L30275

Coral	Bislatumlide A	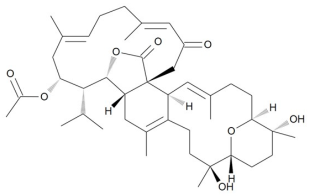	L17993
Coral	Dioxanyalolide	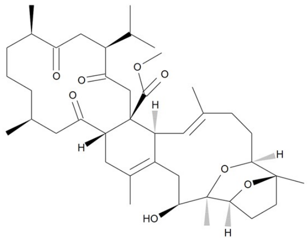	L18401
Coral	Desacetylnyalolide	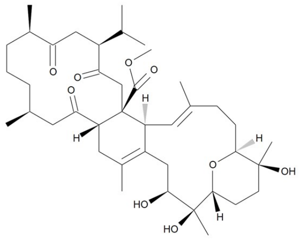	L18399
Coral	Lobophytone W	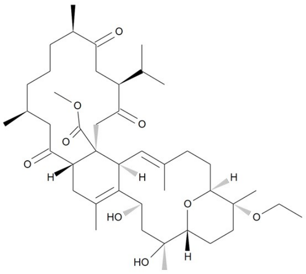	L22576
Coral	24-Methyl-cholesta-5,24(28)-diene-3β,19-diol-7β-monoacetate	Not found	

Coral	Lobohedleolide	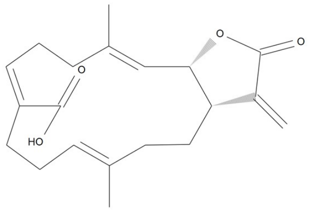	L1632
Coral	Junceellolide D	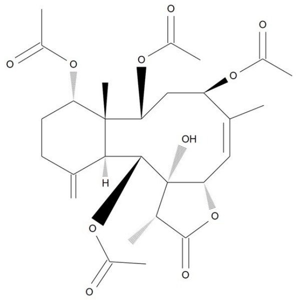	L7525
Coral	Dihydroaustrasulfone	Not found (Synthetic)	
Coral	1-Tosylpentan-3-one	Not found (Synthetic)	
Coral	4-(phenylsulfanyl)butan-2-one	Not found (Synthetic)	
Coral	(1S,2S,3E,7E,11E)-3,7,11,15-cembratetraen-17,2-olide	Not found	
Coral	Pachycladin A	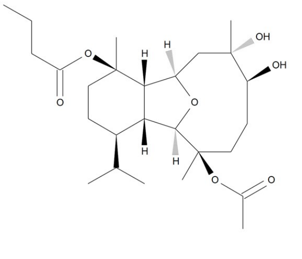	L21083

Coral	Pachycladin B	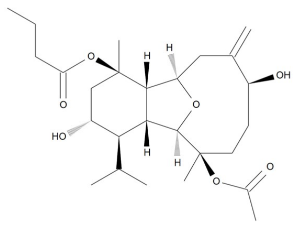	L21084
Coral	Pachycladin C	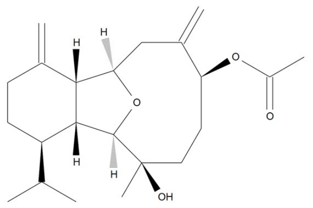	L21085
Coral	Pachycladin D	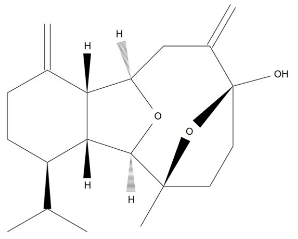	L21086
Coral	Pachycladin E	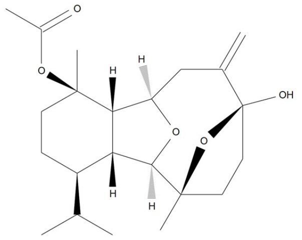	L21087

Coral	Sarcoehrenbergilid A	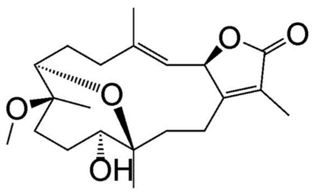	L30612
Coral	Sarcoehrenbergilid B	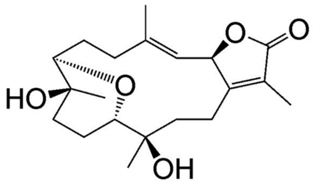	L30613
Coral	Sarcoehrenbergilid C	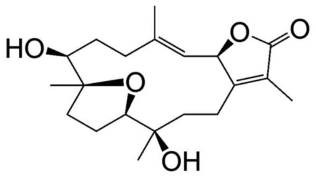	L30614
Coral	Sarcophine	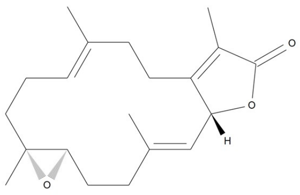	L1842

Coral	(+)-7α-8β-dihydroxydeepoxysarcophine	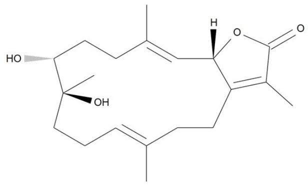	L10839
Coral	Sinulolide A	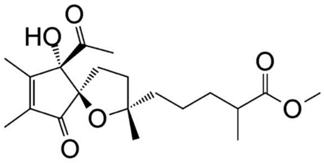	L26872
Coral	Sinulolide B	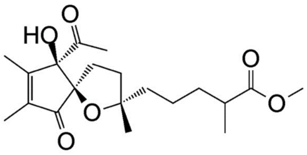	L26873
Coral	Sardisterol	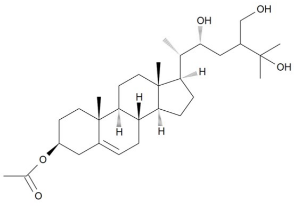	L13077
