# Supplementary material for: Deep-Sea Sponges and Corals off the Western Coast of Florida—Intracellular Mechanisms of Action of Bioactive Compounds and Technological Advances Supporting the Drug Discovery Pipeline
Source: Mar Drugs. 2023 Nov 28;21(12):615. doi: 10.3390/md21120615 (PMC10744787; doi:10.3390/md21120615)
Supplement: Supplementary file 1 [file marinedrugs-21-00615-s001.zip › Supplementary_File_S15.pdf]

Marine Source	Compound	Structure	Compound ID
Sponges	PM050489	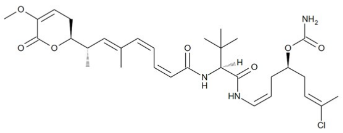	L25150
Sponges	PM060184	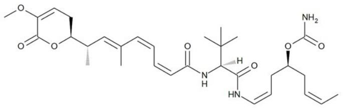	L25151
Sponges	Halichondrin B	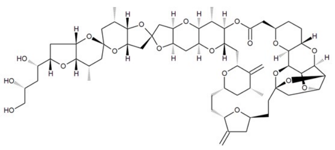	L2471

Sponges	*N*-formyl-7-amino-11-cycloamphilectene	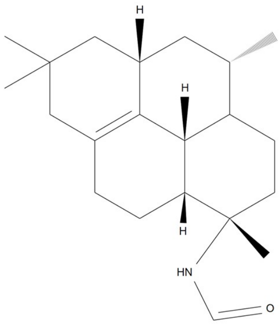	L14384
Sponges	Discodermolide	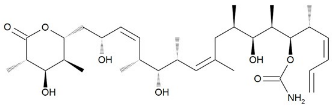	L8745
Sponges	Dictyostatin-1	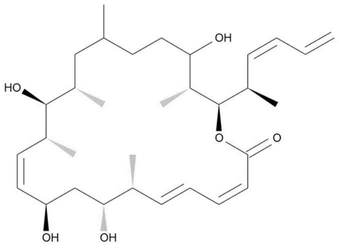	L8431
Sponges	Milnamide A	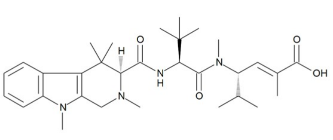	L15496

Sponges	Milnamide B	Not found	
Sponges	Milnamide C	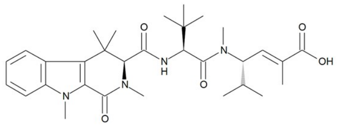	L15495
Sponges	Jaspamide (Jasplakinolide)	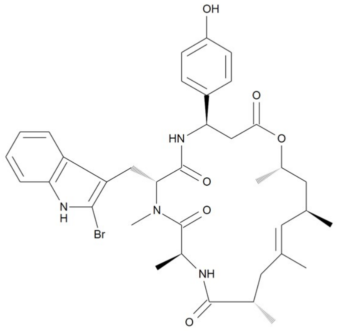	L2316
Sponges	Geodiamolide A	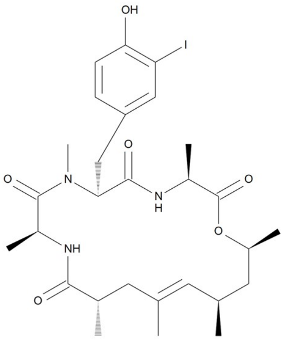	L2617

Sponges	Geodiamolide D	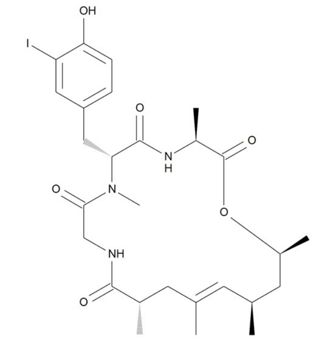	L3471
Sponges	Geodiamolide E	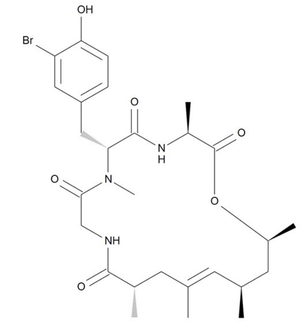	L3472
Sponges	Geodiamolide G	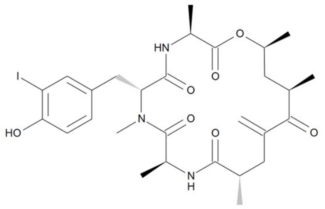	L8560
Sponges	Peloruside E	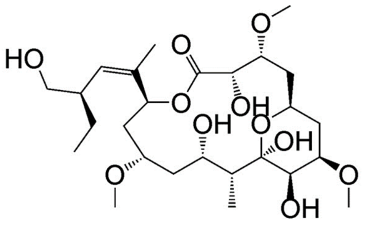	L32494

Sponges	SPIKET-P	Not found (Synthesized)	
Sponges	Spongistatin 1	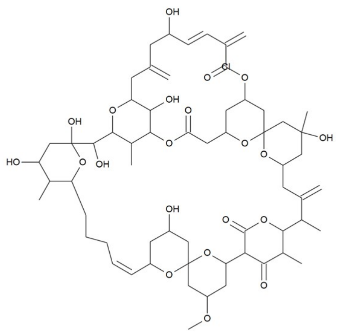	L6097
Sponges	Zampanolide	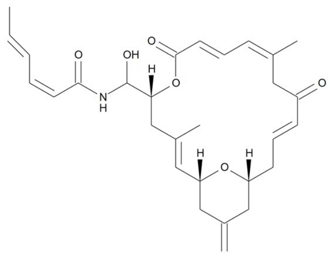	L9236
Sponges	(19Z)-Halichondramide	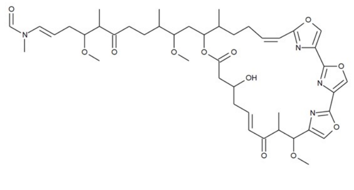	L15662

Sponges	Geodiamolide B	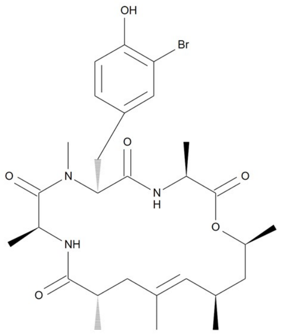	L2618
Sponges	Geodiamolide H	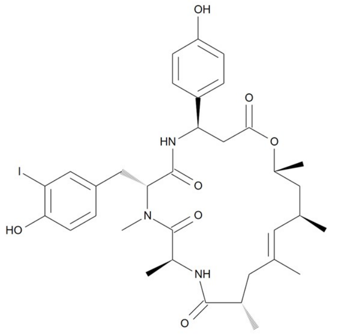	L10414
Sponges	Geodiamolide I	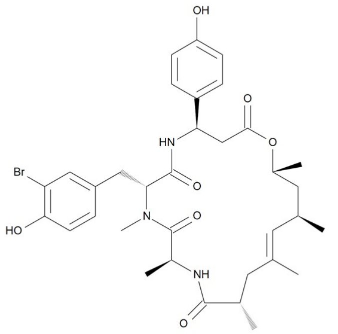	L10415
Sponges	Sphinxolide B	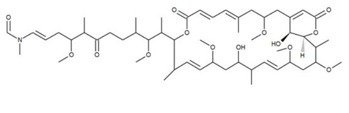	L6720

Sponges	Swinholide I	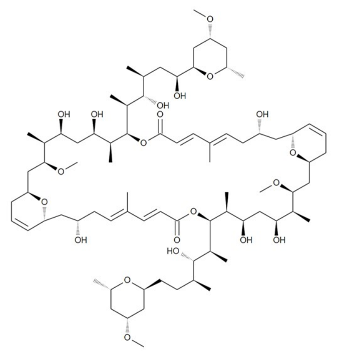	L16748
Sponges	Hurghadolide A	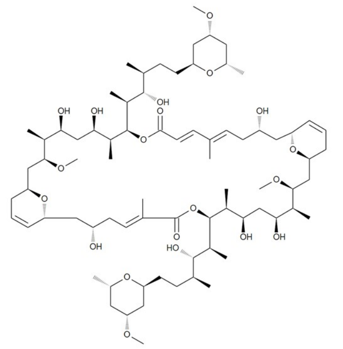	L16749
Sponges	Motuporamine C	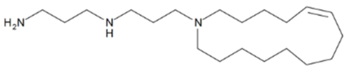	L10660

Sponges	Lasonolide A	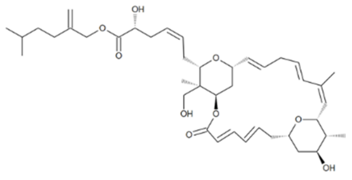	L14334
Sponges	Mycalolide A	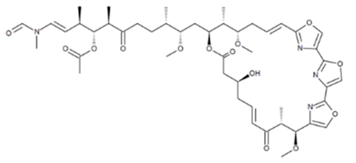	L11589
Sponges	Mycalolide B	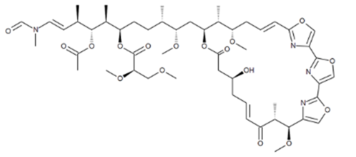	L11590

Sponges	38-Hydroxymycalolide B	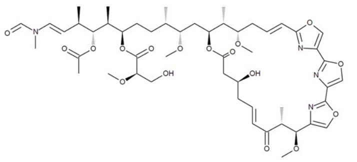	L11594
Sponges	Oxalatrunculin B	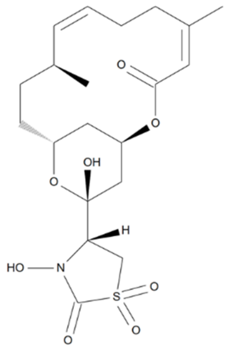	L18202
Sponges	Latrunculin B	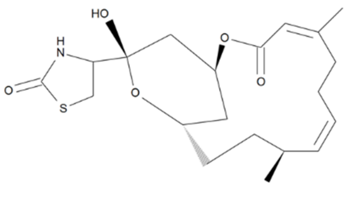	L1935
Sponges	16-epi-latrunculin B	Not found	

Sponges	Latrunculin T	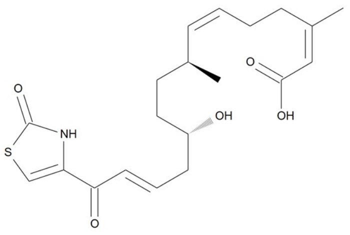	L16785
Sponges	Latrunculin A	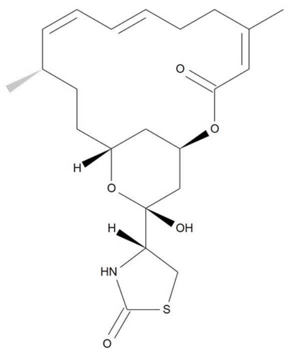	L8806
Sponges	17-O-[N-(phenyl)carbamoyl]-latrunculin A	Not found	
Sponges	17-*O*-phenylethyllatrunculin A	Not found	
Sponges	N-hydroxymethyllatrunculin A	Not found	
Sponges	Kabiramide C	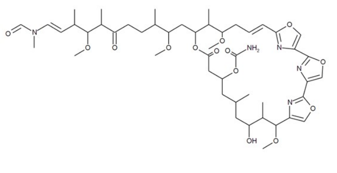	L2518

Sponges	Sceptrin	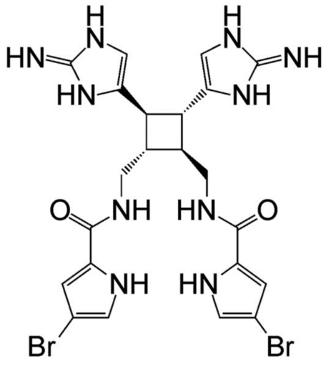	L27082
Sponges	Bromosceptrin	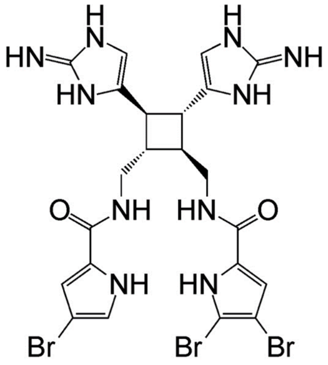	L27083
Sponges	Dibromosceptrin	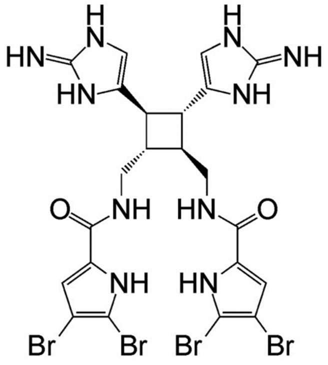	L27084
Sponges	Oxysceptrin	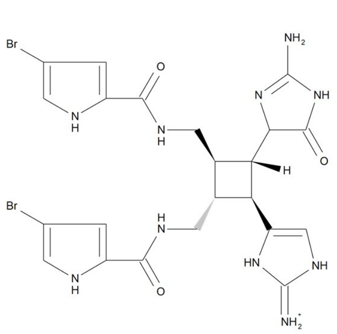	L4900

Sponges	Heteronemin	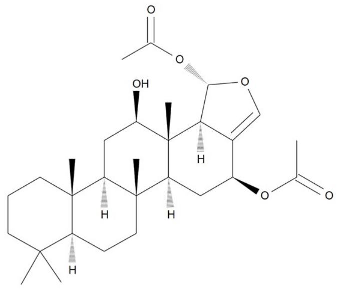	L6940
Sponges	Adociasulfate-2	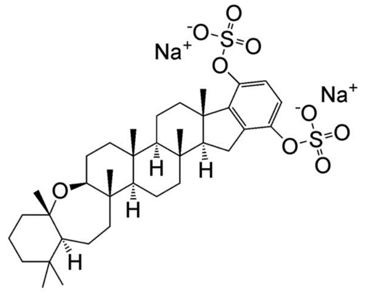	L11550
Sponges	Adociasulfate-13	Not Found	
Sponges	Adociasulfate-14	Not Found	
Sponges	Purealin	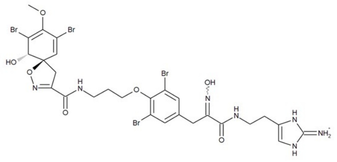	L1997

Sponges	Geoditin A	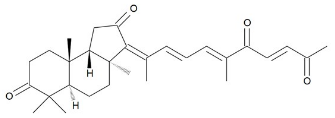	L13404
Sponges	Stellettin A	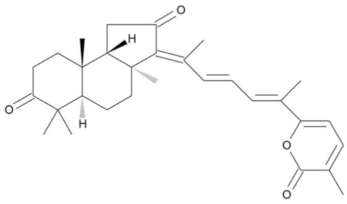	L8009
Sponges	O-Demethylrenierol	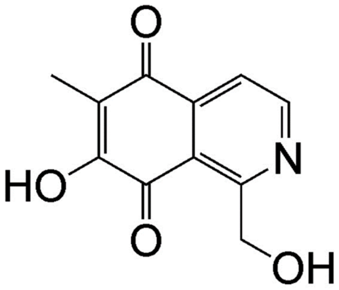	L37088
Sponges	1,6-dimethyl-7-hydroxy-5,8-dihydroisoquinoline-5-8-dione	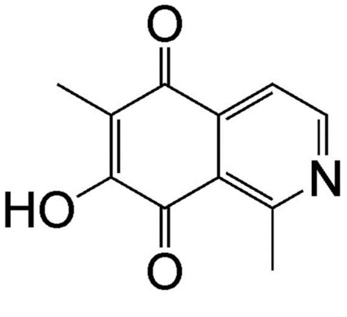	L37089

Sponges	O-demethylrenierone	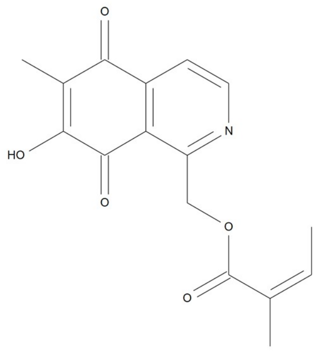	L82
Sponges	Renierone	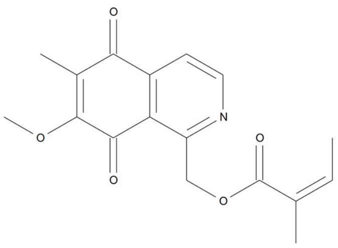	L41
Sponges	N-formyl-1,2-dihydrorenierone	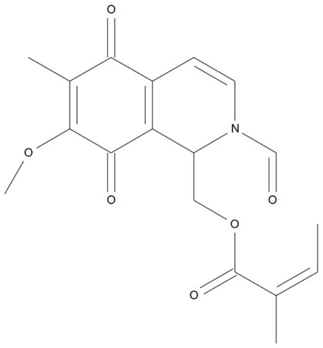	L80

Sponges	Psammaplysene A	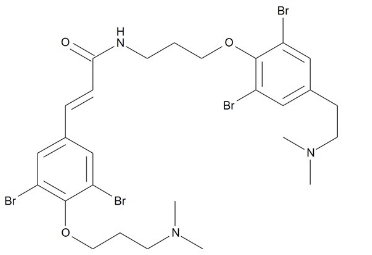	L16046
Sponges	Psammaplysene B	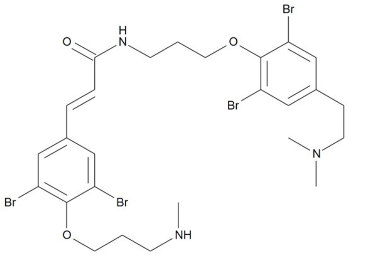	L16047
Sponges	Ilimaquinone	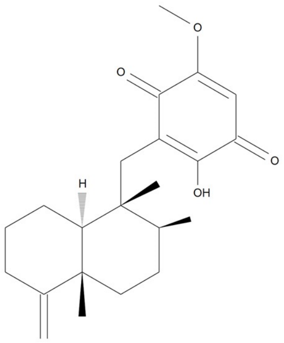	L2724
Sponges	Scalaradial	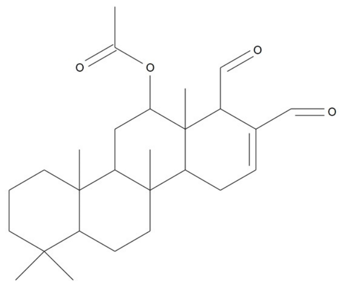	L549

Sponges	Cacospongionolide	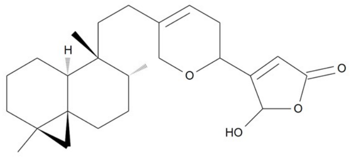	L3033
Sponges	(-)-Agelasidine A	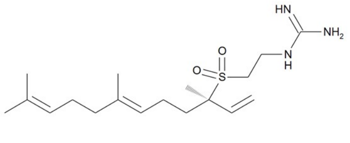	L17336
Sponges	(-)-Agelasine D	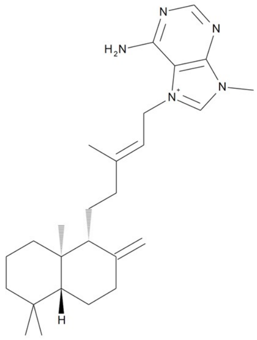	L20711
Sponges	(+)-1(R),6(S),1'(R),6'(S),11(R),17(S)-fistularin-3	Not Found	

Sponges	Isofistularin-3	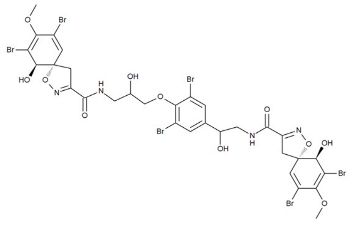	L10619
Sponges	12 β -(3' β -hydroxybutanoyloxy)-20,24-dimethyl-24-oxo-scalara-16-en-25-al	Not Found	
Sponges	Avarol	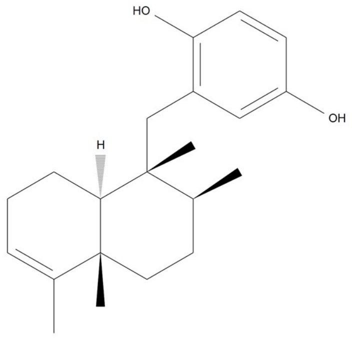	L964
Sponges	6"-Debromohamacanthin A	Not Found	
Sponges	Agelasine G	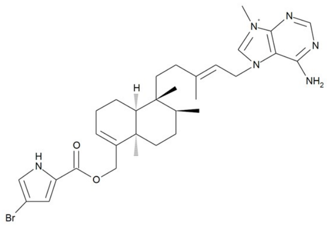	L5622
Sponges	Stellettin B	Not found	

Sponges	Pachatrissamine	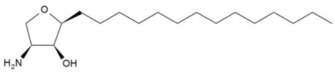	L13863
Sponges	Topsentin	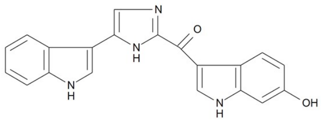	L3851
Sponges	(3S)-icos-4E-en-1-yn-3-ol	Not Found	
Sponges	(3S)-14-methyldocos-4E-en-1-yn-3-ol	Not Found	
Sponges	Crambescidin 800	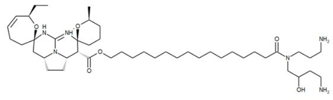	L5324

Sponges	Aurantoside C	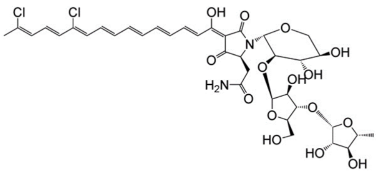	L11243
Sponges	Monanchocidin A	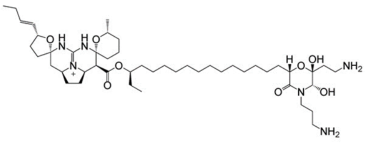	L31943
Sponges	Monanchocidin B	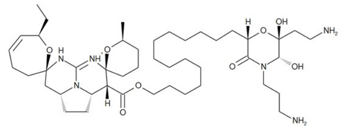	L22537
Sponges	Monanchomycalin C	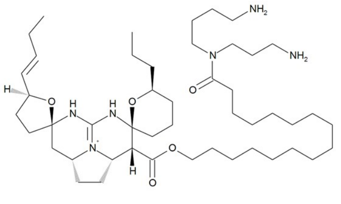	L23849

Sponges	Ptilomycalin A	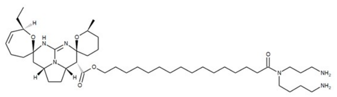	L6120
Sponges	Monanchomycalin B	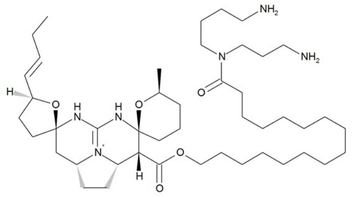	L23850
Sponges	Normonanchocidin D	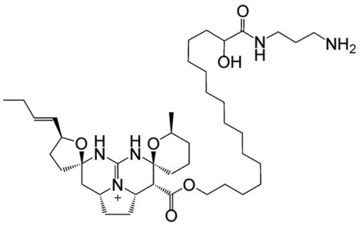	L28330
Sponges	Urupocidin A	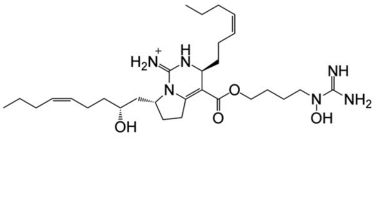	L27009

Sponges	Pulchranin A	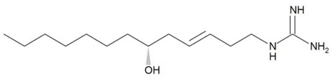	L24611
Sponges	Aldisine	Not found	
Sponges	2-Bromoaldisine	Not found	
Sponges	10Z-debromohymenialdisine	Not found	
Sponges	10E-hymenialdisine	Not found	
Sponges	10Z-hymenialdisine	Not found	
Sponges	Hymenin	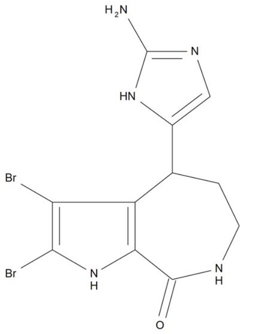	L2414
Sponges	Oroidin	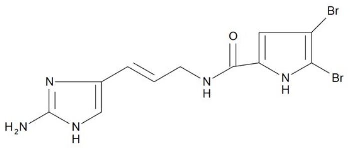	L1051

Sponges	4,5-Dibromopyrrole-2-carbonamide	Not found	
Sponges	Fistularin-1	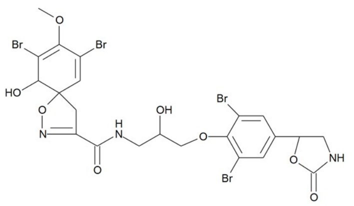	L38
Sponges	Fistularin-2	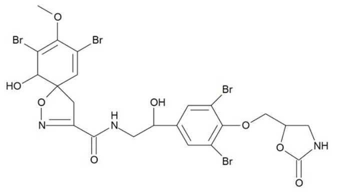	L39
Sponges	Fistularin-3	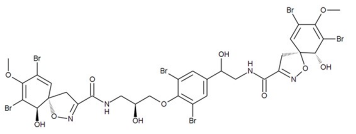	L16179

Sponges	19-Deoxyfistularin-3	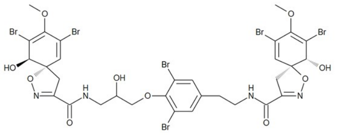	L6526
Sponges	11-Deoxyfistularin-3	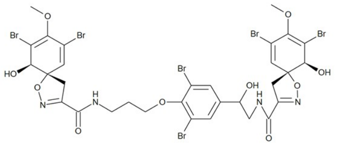	L11867
Sponges	11,19-Dideoxyfistularin-3	Not Found	
Sponges	Jorunnamycin A	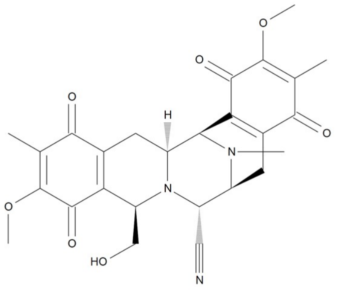	L17571
Sponges	Renieramycin M	Not found	

Sponges	Renieramycin T	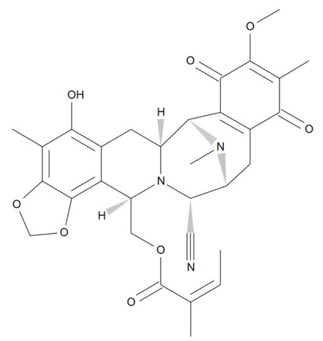	L20025
Sponges	(+)-Liphagal	Not found	
Sponges	Liphagal	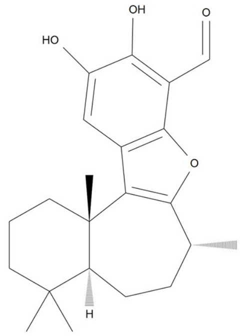	L16682
Sponges	Siphonodictyal B	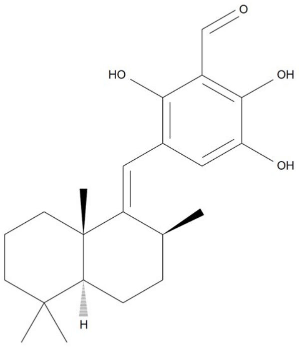	L2407
Sponges	3,7-bis(3,5-dimethylphenyl)-aaptamine	Not found	
Sponges	(+)-Aeroplysinin-1	Not found	

Sponges	Halichondramide	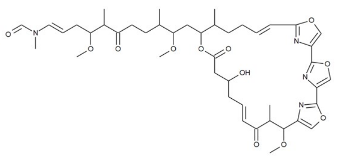	L2830
Sponges	(1’R,5’S,6’S)-2-(3’,5’-dibromo-1’,6’-dihydroxy-4’-oxocyclohex-2’-enyl) Acetonitrile	Not Found	
Sponges	Jaspisamide A	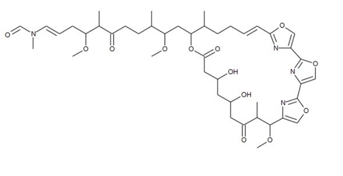	L6235
Sponges	Halishigamide D	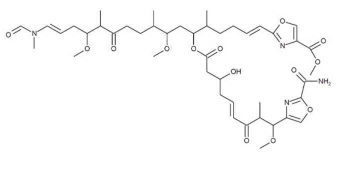	L9492

Sponges	Neohalichondramide	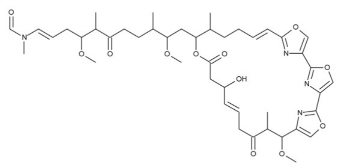	L15661
Sponges	Dysifragilone A	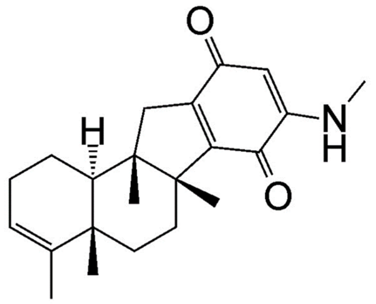	L27208
Sponges	12-Deacetyl-12-epi-scalaradial	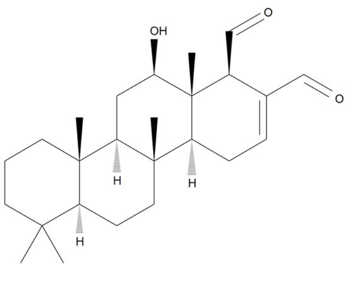	L2403
Sponges	9,11-Dihydrogracilin A	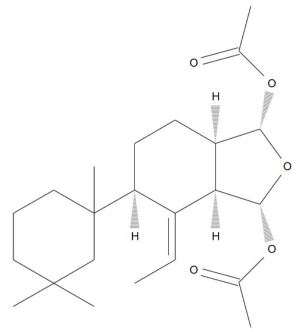	L2450

Sponges	Halenaquinone	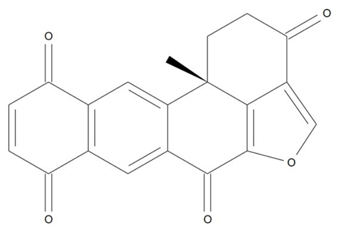	L1120
Sponges	(3R)-icos-(4E)-en-1-yn-3-ol	Not found	
Sponges	(3R)-14-methyldocos-(4E)-en-1-yn-3-ol	Not found	
Sponges	Aaptamine	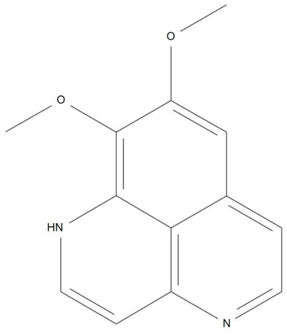	L14595
Sponges	9-Demethyl aaptamine	Not found	
Sponges	Demethyl (oxy)-aaptamine	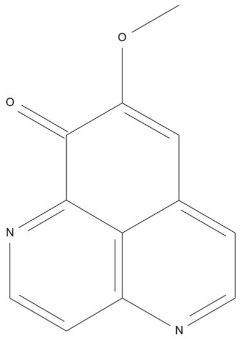	L14597

Sponges	Fascaplysin	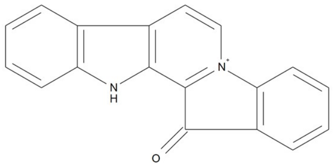	L7361
Sponges	10-Bromo-fascaplysin	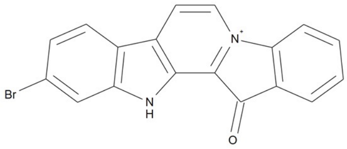	L15129
Sponges	Gracilin J	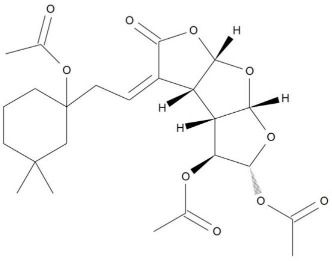	L20172
Sponges	Gracilin K	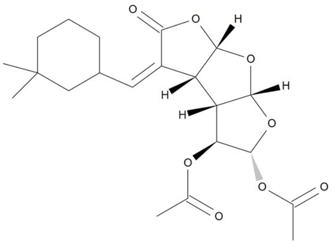	L20173

Sponges	Gracilin H	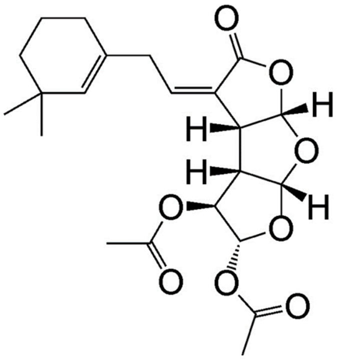	L29370
Sponges	Gracilin I	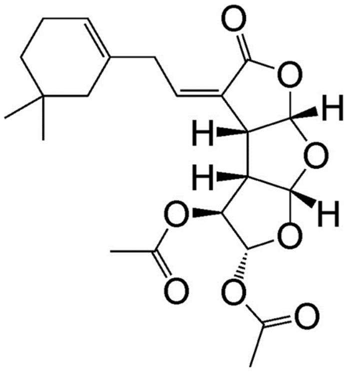	L29371
Sponges	Gracilin L	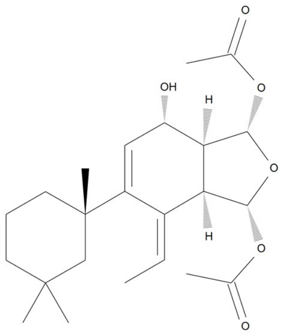	L20174
Sponges	Tetrahydroplysulphyrin-1	Not found	

Sponges	3’-Norspongiolactone	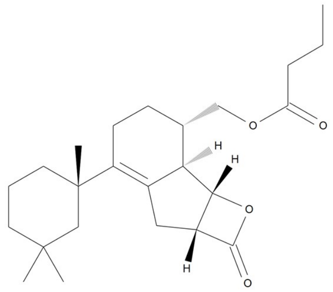	L20175
Sponges	Naamidine A	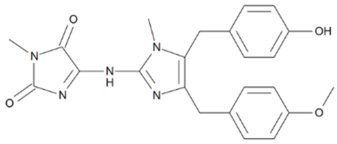	L2704
