# Supplementary material for: Deep-Sea Sponges and Corals off the Western Coast of Florida—Intracellular Mechanisms of Action of Bioactive Compounds and Technological Advances Supporting the Drug Discovery Pipeline
Source: Mar Drugs. 2023 Nov 28;21(12):615. doi: 10.3390/md21120615 (PMC10744787; doi:10.3390/md21120615)
Supplement: Supplementary file 1 [file marinedrugs-21-00615-s001.zip › Supplementary_File_S18.pdf]

LEGENDS FOR SUPPLEMENTARY FILES

Supplementary File 1. PubMed searches performed using search terms relevant to cytoskeleton, protein trafficking, and signaling. The first worksheet presents results for "sea sponge" while the second worksheet presents results for the vernacular sponge names ("demosponge", "calcareous sponge", and "glass sponge").

Supplementary File 2. PubMed searches performed using search terms relevant to cytoskeleton, protein trafficking, and signaling. The first worksheet presents results for "sea coral" while the second worksheet presents results for the vernacular coral names ("soft coral", "stony coral", "cup coral", "gorgonian coral", "branching coral", "black coral", and "lace coral").

Supplementary File 3. PubMed searches performed using search terms relevant to cytoskeleton, protein trafficking, and signaling. The first worksheet presents results for the scientific names for sponges while the second worksheet presents results for the scientific names for corals.

Supplementary File 4. PubMed searches performed regarding technological advances in the cultivation of sponges.

Supplementary File 5. PubMed searches performed regarding technological advances in the cultivation of corals.

Supplementary File 6. NOAA standard data download raw results and analyses. Refer to **Figure 1A – 1C, Figure 2,** and **Table 1**.

Supplementary File 7. NOAA manual data curation results. Results are presented for Quadrant A (refer to **Figure 3A**).

Supplementary File 8. NOAA manual data curation results. Results are presented for Quadrant B (refer to **Figure 3A**).

Supplementary File 9. NOAA manual data curation results. Results are presented for Quadrant C (refer to **Figure 3A**).

Supplementary File 10. NOAA Manual data curation results. Results are presented for Quadrant D (refer to **Figure 3A**).

Supplementary File 11. Classification of corals and sponges. In worksheet 1 and worksheet 2, respectively, data is organized according to phylum, class, subclass, and order. Refer to **Table 1** and **Figure 2**.

Supplementary File 12. Classification of corals for the literature surveyed and analyzed using the WoRMS database.

Supplementary File 13. Classification of sponges for the literature surveyed and analyzed using the WoRMS database.

Supplementary File 14. Marine bioactive compounds derived from corals identified from our literature

survey, as described in Section 4.

Supplementary File 15. Marine bioactive compounds derived from sponges identified from our literature survey, as described in Section 4.

Supplementary File 16. Sponges identified in the technological advances literature review. In the first worksheet, the scientific name, depth of collection, season and year of collection, and relevant details pertaining to the metabolite tested (extract, purified, or synthesized form) are summarized. In the second worksheet, the taxonomical classification of each sponge, as determined through WoRMS (unless otherwise annotated), is summarized.

Supplementary File 17. Corals identified in the technological advances literature review. In the first worksheet, the scientific name, depth of collection, season and year of collection, and relevant details pertaining to the metabolite tested (extract, purified, or synthesized form) are summarized. In the second worksheet, the taxonomical classification of each coral, as determined through WoRMS (unless otherwise annotated), is summarized.
